# Ensemble Docking Coupled to Linear Interaction Energy Calculations for Identification of Coronavirus Main Protease (3CL^pro^) Non-Covalent Small-Molecule Inhibitors

**DOI:** 10.3390/molecules25245808

**Published:** 2020-12-09

**Authors:** Marko Jukič, Dušanka Janežič, Urban Bren

**Affiliations:** 1 Laboratory of Physical Chemistry and Chemical Thermodynamics, Faculty of Chemistry and Chemical Engineering, University of Maribor, Smetanova ulica 17, SI-2000 Maribor, Slovenia; marko.jukic@um.si; 2 Faculty of Mathematics, Natural Sciences and Information Technologies, University of Primorska, Glagoljaška 8, SI-6000 Koper, Slovenia

**Keywords:** COVID-19, SARS-CoV-2, M^pro^, 3CL^pro^, 3C-like protease, virtual screening, inhibitors, in silico drug design, free-energy calculations

## Abstract

SARS-CoV-2, or severe acute respiratory syndrome coronavirus 2, represents a new strain of *Coronaviridae*. In the closing 2019 to early 2020 months, the virus caused a global pandemic of COVID-19 disease. We performed a virtual screening study in order to identify potential inhibitors of the SARS-CoV-2 main viral protease (3CL^pro^ or M^pro^). For this purpose, we developed a novel approach using ensemble docking high-throughput virtual screening directly coupled with subsequent Linear Interaction Energy (LIE) calculations to maximize the conformational space sampling and to assess the binding affinity of identified inhibitors. A large database of small commercial compounds was prepared, and top-scoring hits were identified with two compounds singled out, namely 1-[(R)-2-(1,3-benzimidazol-2-yl)-1-pyrrolidinyl]-2-(4-methyl-1,4-diazepan-1-yl)-1-ethanone and [({(S)-1-[(1H-indol-2-yl)methyl]-3-pyrrolidinyl}methyl)amino](5-methyl-2H-pyrazol-3-yl)formaldehyde. Moreover, we obtained a favorable binding free energy of the identified compounds, and using contact analysis we confirmed their stable binding modes in the 3CL^pro^ active site. These compounds will facilitate further 3CL^pro^ inhibitor design.

## 1. Introduction

Severe acute respiratory syndrome coronavirus 2 or SARS-CoV-2, the pathogen behind the 2019–2020 coronavirus pandemic (COVID-19), is a member of the *Coronaviridae* family, a positive-sense single-stranded (+ssRNA) RNA virus [1,2]. As there are only a handful of therapeutic options for this global threat, novel drug design is critical; thus, we performed a structure-based virtual screening in order to identify potential inhibitors of the main viral protease (3CL^pro^ or M^pro^) of SARS-CoV-2 [3].

In the 3CL^pro^ homodimer, the P1 pocket of the substrate-binding site is formed and seems essential for the catalytically active form [4,5,6]. The enzyme is vital for the processing of coronavirus polyproteins (pp1a, ppa1ab) that are then cleaved by 3C-like and papain-like proteases to form mature non-structural proteins (NSPs), which are themselves involved in subsequent viral replication mechanisms [7]. 3CL^pro^ represents a cysteine protease (EC 3.4.22.69, “3C” refers to the *Picornaviridae* Enterovirus protease 3C) [8] and shares 96% sequence identity with the SARS-CoV main protease (Supporting Information; aligned PDB ID: 6LU7 and 2QIQ with 288 identical residues out of 301) [3,4,8,9]. The substrate recognition pockets in 3CL^pro^ are named as P1–4, and the enzyme is currently the most studied representative in the context of drug design, mainly due to the availability of structural data. X-ray crystal structure of the 3CL^pro^ in complex with the inhibitor N3 has been recently released with PDB IDs 6LU7 and 7BQY at 2.16 and 1.7 Å resolutions, respectively [3]. N3 is a covalent inhibitor of 3CL^pro^, featuring a vinyl carboxyl ester as an electrophilic warhead that acts as a Michael-acceptor, reacting with the catalytic Cys145 nucleophile [3]. Substrate specificity is described as P1-Gln, P2-Leu (hydrophobic), P3-Val (or positively charged residues) or P4-Ala (small hydrophobic), but scientific literature also describes preference for His at the P1 binding pocket of the protease active site [9,10,11,12,13]. Proteolysis itself occurs via a catalytic dyad defined by Cys145 and His41 [14].

**Figure 1:**
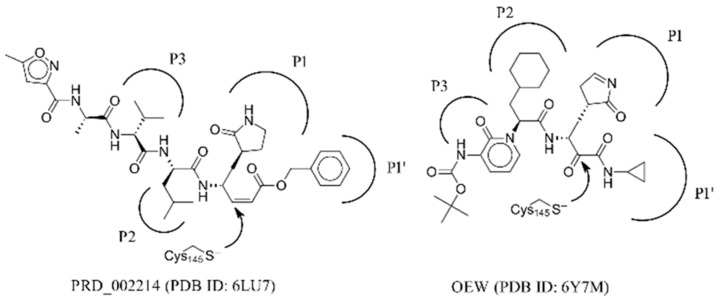
Existing inhibitors of SARS-CoV-2 supported by structural data. Depicted are binding pockets (Px) and the site of covalent reaction.

Considering the currently available structural data, standard in silico docking efforts towards novel potential inhibitors of SARS-CoV-2 main protease are underway [15]. However, only two peptide-like covalent inhibitors have been reported in scientific literature [3]. Due to drawbacks associated with covalent inhibitors, we opted for the identification of novel non-covalent protease inhibitors in a robust screening experiment [16]. We believe the non-covalent inhibitors offer synthetic availability, the flexibility of optimization and can also be used for the future design of covalent inhibitors, if necessary [17]. To this end, we developed a novel methodology directly coupling ensemble docking high-throughput virtual screening (HTVS) with subsequent Linear Interaction Energy (LIE) calculations. Ensemble docking affords viable starting ligand poses and ensemble protein conformations, thereby maximizing the conformational space sampling and yielding reliable ligand binding affinities in the following LIE step. To the best of our knowledge, only SARS-CoV 3CL^pro^ small-molecule inhibitors are reported in the scientific literature and can be used as starting points, but no SARS-CoV-2 3CL^pro^ small-molecule non-covalent inhibitors are available as of yet (Figure 1) [18].

## 2. Results and Discussion

### 2.1. Database Preparation

**Figure 2:**
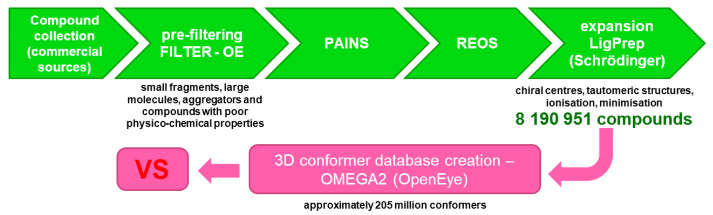
Database preparation for subsequent virtual screening (VS) on the SARS-CoV-2 main protease 3CL^pro^ or M^pro^. The final database contained 8,190,951 molecules before conformer generation.

In a contemporary VS (virtual screening) or HTVS (high-throughput virtual screening) scenario, database design is essential for efficient CPU-time usage in downstream calculations. In order to commence a robust HTVS scenario, we gathered commercially available databases (e.g., ENAMINE, Vitas-M, Chembridge, Maybridge, Ambinter, Otava, PrincetonBIO, Key-Organics, Life Chemicals, Uorsy, Specs) and pre-filtered all compounds in order to exclude small fragments or extra-large molecules, aggregators, and compounds with poor physico-chemical properties. This step was performed using OpenEye FILTER software (OpenEye Scientific Software, Inc., Santa Fe, NM, USA; www.eyesopen.com). The following parameters were used: min_molwt 250, max_molwt 800, min_solubility moderately, eliminate known and predicted aggregators and allowed elements H, C, N, O, F, S, Cl, Br, I and P. This database was subsequently filtered for PAINS [19,20,21] and REOS structures in order to eliminate reactive and labile functional groups [22,23]. For this step we used KNIME software with RDKit software nodes to compare all structures in the library to the selection of SMARTS-formatted flags and to remove hits from the database. We ended up with a collection of approximately 4 million compounds that was expanded in the subsequent step where final enumeration of undefined chiral centers, tautomeric structures, removal of structural faults, ionization at the pH of 7.4 and minimization (using OPLS 3 force-field) towards the final 3D conformation was performed. For this work, Ligprep tool by Schrödinger (Release 2018–3, Schrödinger, LLC, New York, NY, USA 2020) was employed [24,25]. The final database thus consisted of 8,190,951 molecules and was ultimately used for conformer 3D-database preparation using OpenEye OMEGA2 tool (OpenEye Scientific Software, Inc., Santa Fe, NM, USA; www.eyesopen.com). A maximum number of conformations was set at 25, and rms threshold of 0.8 nm afforded approximately 205 million compound conformations ready for VS (Figure 2).

### 2.2. Target Preparation

**Figure 3:**
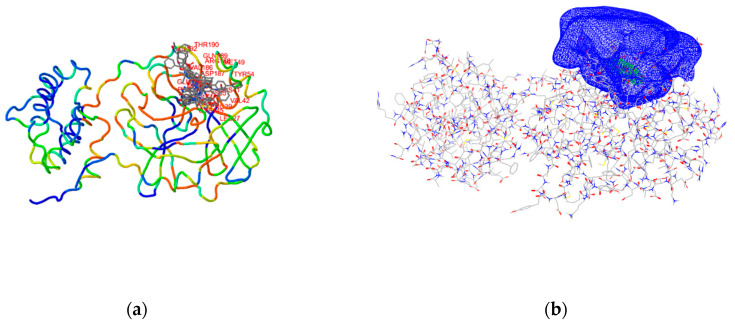
(**a**) Identification of the active site in the vicinity of the Cys145 using the ProBiS server and superposition of the small-molecule ligands from the locally aligned complexes at the 3CL^pro^ active site; (**b**) the resulting extended docking grid calculated by Make Receptor of the OpenEye software.

Next, we examined the available experimental SARS-CoV-2 3CL^pro^ crystal structures and identified the main protease in complex with N3 peptide-like covalent inhibitor published by Yang, H. et al. (PDB ID: 6LU7) [3]. The ligand possessed a Michael-acceptor as an electrophilic warhead to react with the active site cysteine. The covalent bond was cleaved, the N3 residue removed, the cysteine amino-acid residue regenerated (Open Source PyMOL, release 2.1) and the target prepared using Schrödinger Small-Molecule Discovery Suite (Schrödinger LLC, New York). Residue conformation at the active site was checked by superposition with PDB ID: 6M2N with an all atom RMSD of 0.538 Å [26]. As our main goal represented the identification of potential 3CL^pro^ non-covalent inhibitors, we did not want to limit the VS search to a binding pocket occupied by the only reported N3 inhibitor. Thus, we examined similar protein complexes published in the PDB database using ProBiS server (https://probis.nih.gov/) to identify all possible small-molecule binding modes in the vicinity of the catalytic Cys145 (Figure 3). Therefore, a PDB ID: 6LU7 3CL^pro^ was used as an input for ProBiS calculation and one binding site identified (binding site 1 in ProBiS; proximity of Cys145). ProBiS server thus produced local superimposition on the defined binding site, and the ligands from the locally superimposed proteins with structural data (PDB IDs: 2op9, 2gz8, 4mds, 3v3m, 4twy, 3vb4, 2hob, 3tnt, 3vb6, 2gx4 and 2gtb) were used for extended receptor space definition. [27,28].

The postulated binding site, located in the proximity of Cys145, was furnished with several superimposed ligands from locally aligned similar protein structures (PDB IDs: 2op9, 2gz8, 4mds, 3v3m, 4twy, 3vb4, 2hob, 3tnt, 3vb6, 2gx4 and 2gtb), which along with the N3-3CL^pro^ crystal complex (PDB entry: 6LU7) served the subsequent docking definition. With the binding site defined, the receptor structure was generated using OEDocking 3.2.0.2 software package (OpenEye Scientific Software, Inc., Santa Fe, NM, USA; www.eyesopen.com; details are in Supporting Information).

### 2.3. Ensemble Docking

**Figure 4:**
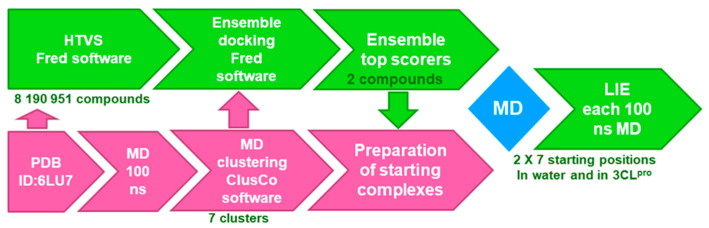
HTVS workflow incorporating ensemble docking coupled to LIE calculations initiated from multiple MD ensemble complexes for the two top-scoring compounds.

For the HTVS step, we performed a robust ensemble docking experiment coupled to a subsequent Linear Interaction Energy (LIE) calculation workflow into the prepared receptor binding site to afford 1‰ of top-scoring compounds, as depicted in Figure 4 (Fred; OpenEye Scientific Software, Inc., Santa Fe, NM, USA; www.eyesopen.com).

In the next step, 3CL^pro^ ensemble was prepared via a 100 ns molecular dynamics (MD) simulation (PDB ID: 6LU7) followed by a pairwise average-linkage Hierarchical clustering step (ClusCO software, clustering considered the backbone atoms of the whole target protein) to afford 7 representative protein conformations [29,30]. Using OEDocking software (3.2.0.3, OpenEye Scientific Software, Inc., Santa Fe, NM, USA; www.eyesopen.com), mean FRED scores from the ensemble docking experiment highlighted top-scoring compounds with the top five compounds **1** to **5** collected in Table 1 and Table S5 [31,32,33,34]. FRED scores for the top 100 scoring hits resided below −12.1 with the top-scorer **1** at −14.5, compound **2** at −13.5 and all other hits above −13.0. No molecular weight or cLogP FRED score bias could be observed for the top-scoring compounds, so no additional docking score normalization step had to be performed (Supporting Information, Figures S19–S23). Ensemble docking was somewhat analogous to a classical docking experiment with all residues in the active site kept flexible during the docking runs. Our objective was to verify the predicted binding poses and to postulate that the compounds can adopt a favorable binding mode even with protein flexibility considered as opposed to performing a classical docking experiment with a single rigid protein conformation (Supporting Information, Figure S18).

### 2.4. Free-Energy Calculations and Contact Analysis

**Figure 5:**
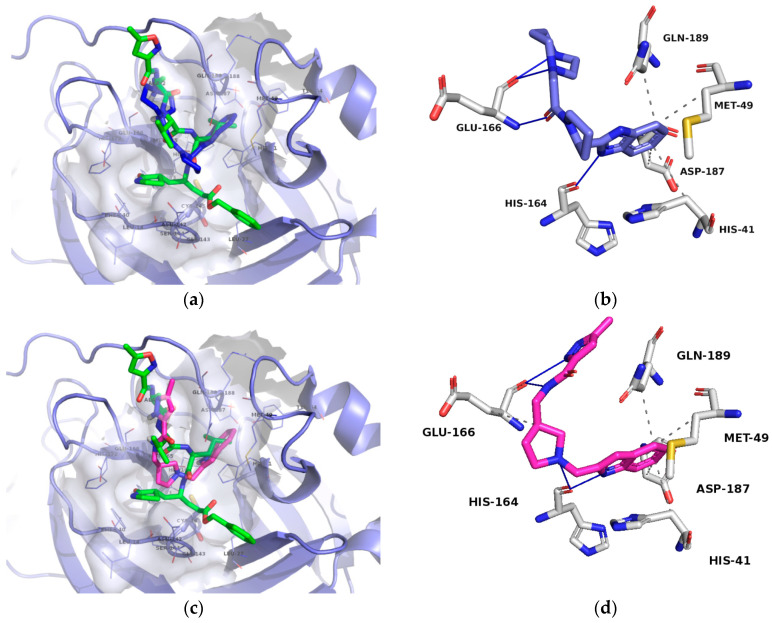
(**a**): Compound **1** binding mode presented in blue colored stick model; (**b**): Compound **1** key interactions; (**c**): Compound **2** binding mode presented in cyan colored stick model. Reference N3 ligand from the PDB ID: 6LU7 is depicted in green-colored stick model and the protein surface around the ligand calculated. (**d**): Compound **2** key interactions. Hydrogen bonds are denoted as blue lines and hydrophobic interactions as grey lines.

The Linear Interaction Energy (LIE) method was applied for calculating the binding free energy of the identified two top-scoring compounds [35,36]. Compounds **1** and **2** with the FRED scores of −14.5 and −13.5, respectively, displayed significantly lower values (below −13.5) and were followed by 7 compounds with scores in the narrow range of −13.0 to −12.8. Compounds **1** and **2** were, therefore, selected for subsequent LIE calculation based on the predicted docking score and a favorable binding pose generation. The docked conformations of the two top-scoring compounds exhibited an analogous positioning in the S2–S3 pocket of the 3CL^pro^ active site interacting mainly with His41, Met49, His164, Glu166, Asp187 and Gln189 (Figure 5) [37]. The predicted binding mode is in accordance with the crystal ligand N3 (PDB ID: 6LU7) and analogous to the reported binding mode of a potential SARS-CoV-2 3CL^pro^ inhibitor dipyridamole reported by Li et al. [38]. Othar et al. identified similar interaction profiles upon MD experiments using FDA-approved compounds [39].

Clustered complexes from the ensemble docking were used as starting ligand-receptor bound complexes, i.e., 7 starting complexes for each compound, subjected to the 100 ns MD production run along with corresponding 100 ns MD simulations in ligand-free state for maximal conformational space coverage. Weighted LIE calculation demonstrated the affinity towards 3CL^pro^ for compound **1** with _Δ_G_LIE-BIND_ value of −8.2 ± 1.9 and for compound **2** with _Δ_G_LIE-BIND_ value of −3.5 ± 1.7 kcal/mol using pre-optimized α and β parameters (Table 2). Moreover, ligand-protein contact analyses along all 7 production runs of compound **1** confirmed the hydrogen bond formation with Glu166, Asp187, Gln189, His41 (for more than 90% of simulation time) and His164 (for more than 50% of simulation time) along with an average of 7 hydrophobic contacts along all 100 ns production runs. Similar observations in all MD simulation runs were made for compound **2** as well. Hydrogen bonds with Gln192, Glu166, Gln189, His164 (for more than 90% of simulation time), Glu166, Val186, Arg188 and Thr190 (for more than 50% of simulation time) along with an average of 9 hydrophobic contacts were formed (details on individual MD replicas can be found in Supporting Information, Figures S2–S15). MD simulations thus place compounds **1** and **2** near P1, tightly into the P2–P3 pockets of the 3CL^pro^ active site in a close proximity (under 4 Å) to the catalytic Cys145 and His41 residues.

## 3. Materials and Methods

### 3.1. MD and Ensemble Docking

Crystal complex (PDB ID: 6LU7) was prepared with Yasara software [29]. Missing hydrogens were added, overlapping atoms adjusted, hydrogen bonds optimized and residue ionization assigned at pH = 7.4 [40,41]. The system was solvated using TIP3P (cubic; 10 Å padding, periodic boundary conditions applied) water model, and a physiological concentration of 0.9% of NaCl ions was added with an appropriate excess of either Na^+^ or Cl^-^ to neutralize the cell (long-range Coulomb forces calculated using particle-mesh Ewald algorithm). After steepest descent and simulated annealing minimizations to remove sterical clashes, the MD simulation was run for 100 ns using the AMBER14 force field for the protein, GAFF for the ligands as well as TIP3P for water [42,43]. Ligand charges were assigned using AM1-BCC [44]. The equations of motion were integrated with a multiple timestep of 1.25 fs for bonded interactions and 2.5 fs for non-bonded interactions at a temperature of 298K and a pressure of 1 atm (NPT ensemble) using algorithms described in detail previously with snapshots saved every 100 ps [45]. Hydrogen atom bonds were not constrained during the simulation. Energy parameters of the system were stable throughout the production run as were root-mean-square deviation (RMSD) values of protein backbone. Protein conformation models (MD snapshots, 100 structures) were aligned using Theseus and iteratively clustered using ClusCo software (hierarchical clustering, pairwise average-linkage manner, rmsd score) in order to obtain clusters covering a representative portion of the trajectory (with all cluster occupancies above 1% and no single-structure clusters formed) [30,46]. We identified 7 clusters and centroid structures were selected for target preparation as described in the previous chapter.

Ensemble docking was performed using FRED software and the final score calculated as the median FRED score (7 docking experiments on 7 identified protein conformations). Docked complexes of the top-scoring compounds **1** and **2** (Table S5) in all 7 target conformations from ensemble docking were used in subsequent LIE calculations. We generated 14 independent 100 ns MD trajectories totaling to 1.4 µs of simulation time for each ligand in protein and water environments in order to thoroughly explore the conformational space of the system. Moreover, the starting coordinates of ligands and ligand–protein complexes were as varied as possible due to preceding ensemble docking step, thereby effectively enhancing the conformation space sampling.

Simulations of free ligands in water were performed as well to estimate potential energies for calculating binding affinities using LIE methodology. Standard MD simulations were run on a ligand in TIP3P water. MD simulations of 100 ns were carried out analogously as described above for receptor–ligand complexes (no restraints were applied). Based on generated snapshots (1000 snapshots per run), we ran MD energetics analyses to get the VDW and electrostatic interactions of the ligands, and their average values were taken for the subsequent LIE calculation.

### 3.2. Free Energy Calculation Using LIE Methodology

A number of different computational approaches are available for predicting or estimating binding free energies. Linear Interaction Energy (LIE) methodology was proposed by Aqvist et al. [47]. This is a semiempirical method based on the linear response theory. This methodology focuses on the starting and end states of the binding process, which are the free and the bound state of the ligand. LIE is usually less computationally intensive than FEP but on the contrary with the popular MM-PB(GB)SA, uses an explicit solvent model; therefore, de-solvation can be handled in an explicit manner. The concept of the LIE approach is to separately calculate the VdW and electrostatic interaction energies of the ligand in water and of the ligand in complex with solvated protein. Then, average interaction energies between the ligand and its surroundings are analyzed using Equation (1).

(1) $${\Delta G}_{bind}^{}=\;\alpha \;\Sigma_{i}^{N}{\;\Delta V}_{i}^{\mathrm{VdW}}+\beta \;\Sigma_{i}^{N}\;{\Delta V}_{i}^{coulomb}$$

In Equation (1) the ∆ term indicates the change in potential energy between the ligand bound and ligand free (in water) states. The α and β represent LIE empirical parameters, determined by comparing calculated and experimentally measured binding affinities. Their values are optimized by Aqvist et al. [47].

For the LIE approach, the MD simulations of 100 ns of receptor-bound ligands (7 complexes for 2 ligands, 14 in total) as well as of free ligands in water were carried out to obtain the VdW and electrostatic interaction energies between the ligand and its surroundings. Each MD simulation afforded 1000 snapshots (using 100 ps intervals) of each compound in different environments which were used for calculating average electrostatic and VdW energies. Energies were simply averaged for the ligand-free simulation (2 sets separately for compounds **1** and **2**), while ligand bound potential energies were initially weighted according to Equation (2) and subsequently used in the general LIE Equation (1) (Table 2) [35,36,48]. The employed method was reported as suitable for calculation of ligand binding free energy, compared to MM/PBSA and reported to produce relevant results even with shorter simulation times when compared to alternative methodologies [49].

(2) $$W_{i}^{}=\;\frac{e^{-{\Delta G}_{calc,i\;}^{}/k_{b}^{}{T\;}_{}^{}}}{\Sigma_{i}^{}e^{-{\Delta G}_{calc,i\;}^{}/k_{b}^{}{T\;}_{}^{}}}$$

## 4. Conclusions

This work presents an extensive and robust virtual screening scenario on the SARS-CoV-2 main protease 3CL^pro^ or M^pro^, a potential therapeutic target for COVID-19. We report two top-scoring compounds, 1-[(R)-2-(1,3-benzimidazol-2-yl)-1-pyrrolidinyl]-2-(4-methyl-1,4-diazepan-1-yl)-1-ethanone and [({(S)-1-[(1H-indol-2-yl)methyl]-3-pyrrolidinyl}methyl)amino](5-methyl-2H-pyrazol-3-yl)formaldehyde, as viable binders supported by LIE calculations starting from multiple ensemble 3CL^pro^ conformations. This is an in silico work that warrants experimental support in future research and, if successful, the presented set of compounds could be validated for further development of novel non-covalent inhibitors of SARS-CoV-2 main protease (or be used as probes or experimental decoys). Last but not least, the reported hits possess a relatively low molecular weight, their scaffolds are suitable for synthetic optimization and should be synthetically accessible and even commercially available.

Moreover, the presented methodology where ensemble docking is used to identify viable starting ligand poses and ensemble protein conformations directly coupled to subsequent Linear Interaction Energy (LIE) calculations represents a novel advantageous approach to maximize the conformational space sampling thereby facilitating reliable ligand binding free energies.

## Figures and Tables

**Table 1:** Identified top-scoring compounds in the ensemble docking VS on the SARS-CoV-2 main protease (for the complete list see Table S5 in the Supporting Information).

No.	Structure	Mr (g/mol)	Binding Mode	Fred Docking Score ^1^
1	* 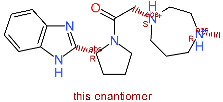 *	343.5	P2–P3	−14.5
2	* 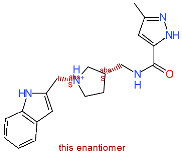 *	338.4	P2–P3	−13.5
3	* 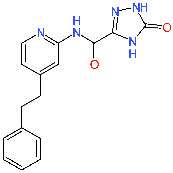 *	309.3	P2–P3	−13.0
4	* 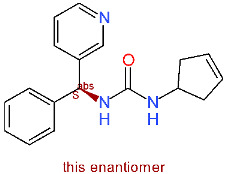 *	293.4	P1′–P2	−13.0
5	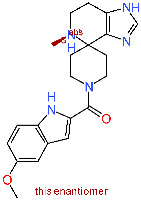	380.5	P1′–P2	−13.0

^1^ Cleaved native ligand N3 from PDB ID: 6LU7 served as a re-docking validation.

**Table 2:** Calculation of binding free energies for compounds **1** and **2** using LIE methodology.

Compound	Free VdW (kcal/mol)	Free Coulomb (kcal/mol)	Complex VdW Weighted Sum (kcal/mol)	Complex Coulomb Weighted Sum (kcal/mol)	$\Delta G_{LIE}^{BIND}$ (kcal/mol)
**1**	−16.2 ± 0.2	−32.3 ± 0.1	−22.0 ± 1.4	−37.3 ± 2.4	−8.2 ± 1.9
**2**	−14.7 ± 0.2	−19.0 ± 0.1	−22.5 ± 2.4	−18.7 ± 2.6	−3.5 ± 1.7

## Supplementary Materials

The following are available online. Details on library and target preparation, sequence alignment, virtual screening, MD clustering, contact analysis as well as on top hit compounds (Figures S1–S24, Tables S1–S5).

## Abbreviations

HTVS	High-Throughput Virtual Screening
LIE	Linear Interaction Energy
MD	Molecular Dynamics
M^pro^	Main Protease
PAINS	Pan-assay interference compounds
REOS	Rapid elimination of swill filter
VS	Virtual Screening
