# Supplementary material for: Ensemble Docking Coupled to Linear Interaction Energy Calculations for Identification of Coronavirus Main Protease (3CLpro) Non-Covalent Small-Molecule Inhibitors
Source: Molecules. 2020 Dec 9;25(24):5808. doi: 10.3390/molecules25245808 (PMC7763084; doi:10.3390/molecules25245808)
Supplement: Supplementary file 1 [file molecules-25-05808-s001.pdf]

molecules

Supporting Information

Ensemble Docking Coupled to Linear Interaction Energy Calculations for Identification of Coronavirus Main Protease ($3CL^{pro}$) Non-Covalent Small-Molecule Inhibitors

Marko Jukič [1,2], Dušanka Janežič [2,*] and Urban Bren [1,2,*]

[1] Laboratory of Physical Chemistry and Chemical Thermodynamics, Faculty of Chemistry and Chemical Engineering, University of Maribor, Smetanova ulica 17, SI-2000 Maribor, Slovenia.

[2] Faculty of Mathematics, Natural Sciences and Information Technologies, University of Primorska, Glagoljaška 8, SI-6000 Koper, Slovenia.

* Correspondence: Email: dusanka.janezic@upr.si (D.J.); urban.bren@um.si (U.B.)

molecules

1. Library preparation and pre-filtering criteria

Pre-filtering of compounds was performed with OpenEye FILTER software and exact parameters used were: min_molwt 250, max_molwt 800, min_solubility moderately, eliminate known and predicted aggregators and allowed elements: H,C,N,O,F,S,Cl,Br,I,P.

Small molecules with nonspecific binding potential have been removed by min_molwt 250 parameter while large molecules with poor optimisation potential were removed with max_molwt 800 parameter. Compounds have been filtered according to favourable XlogP using min_solubility moderately parameter. The solubility predictions are based on using the atom-types from the XLogP algorithm, and reparameterizing them based on available solubility data (The 6 allowable categories are: insoluble**,** poorly**,** moderately**,** soluble**,** very**,** highly). [1]

Furthermore known and predicted aggregators were removed using AGGREGATORS true "Eliminate known aggregators" and PRED_AGG true "Eliminate predicted aggregators" parameters.

Aggregators are small molecules that can interfere with assay results by sequestering protein in an aggregation of small molecules in solution. They appear to have activity in many assays, but in fact are usually not specific inhibitors of the protein in question. Includes two measures of whether a molecule is one of the aggregators defined by Shoichet et. al. The first measure, AGGREGATORS, is whether the molecule is an exact match to one of the approximately 400 published aggregators. The second measure, PRED_AGG, is whether the molecule hits in Shoichet's QSAR model for predicting aggregators. [2, 3]

Compounds were also filtered if they contained other elements than H,C,N,O,F,S,Cl,Br,I,P in order to filter out metal complexes and reactive compounds.

2. TARGET PREPARATION

On examination of SARS-CoV-2 3CL^{pro} complexes, we identified a main protease in complex with N3 peptide-like covalent inhibitor ((phenylmethyl) (4~{S})-4-[[(2~{S})-4-methyl-2-[[(2~{S})-3-methyl-2-[[(2~{S})-2-[(5-methyl-1,2-oxazol-3-yl)carbonylamino]propanoyl]amino]butanoyl]amino]pentanoyl]amino]-5-[(3~{S})-2-oxidanylidenepyrrolidin-3-yl]pent-2-enoate;

molecules MDPI

SMILES: CC(C)C[C@H](NC(=O)[C@@H](NC(=O)[C@H](C)NC(=O)c1cc(C)on1)C(C)C)C(=O)N[C@@H](C[C@@H]2CCNC2=O)\C=C/C(=O)OCc3ccccc3) published by Liu, X *et al*. (PDB ID: 6LU7). N3 inhibitor was found at the active site and was covalently bound to the catalytic Cys145 residue. Covalent bond was cleaved, the N3 residue was removed and the cysteine amino-acid residue regenerated (Open Source PyMOL, release 2.1). Using Schrödinger Small-Molecule Discovery Suite (release 2018-3, Schrödinger LLC, New York) the missing hydrogen atoms were added, steric clashes corrected and water molecules deleted. Protonation and optimization of sidechains within the protein was performed using the PROPKA tool (Schrödinger at pH = 7.4) and the resulting structure used for similarity search. To expand the binding pocket, we examined similar protein complexes reported in the PDB database using ProBiS Charmming server (https://probis.nih.gov/). [4-6] This identified one selective binding site (binding site 1; proximity of Cys145) and several ligands from the locally aligned (relative to the binding site 1) similar protein structures were superimposed on the examined complex (PDB IDs: 2op9, 2gz8, 4mds, 3v3m, 4twy, 3vb4, 2hob, 3tnt, 3vb6, 2gx4 and 2gtb) (Figure 3, main-text).

ProBiS output on binding site 1 (the only predicted selective active site). Due to redundancy, 10 first ligands were used along with the N3 for binding site definition:

Type Molecule Name Residue Name Source Confidence Binder

small molecules

Binding Site 1

NALPHA-[(BENZYLOXY)CARBONYL]-N-[(1R)-4-HYDROXY-1-METHYL-2-OXOBUTYL]-L-PHENYLALANINAMIDE WR1 2op9 4.34 Specific

S-[5-(TRIFLUOROMETHYL)-4H-1,2,4-TRIAZOL-3-YL] 5-(PHENYLETHYNYL)FURAN-2-CARBOTHIOATE F3F 2gz8 4.34 Specific

BENZENE BNZ 2z9g 4.34 Specific

N-[4-(ACETYLAMINO)PHENYL]-2-(1H-BENZOTRIAZOL-1-YL)-N-[(1R)-2-[(2-METHYLBUTAN-2-YL)AMINO]-1-(1-METHYL-1H-PYRROL-2-YL)-2-OXOETHYL]ACETAMIDE 23H 4mds 4.34 Specific

DIMETHYL SULFOXIDE DMS 2z9j 4.34 Non-specific

N-[(1R)-2-(TERT-BUTYLAMINO)-2-OXO-1-(PYRIDIN-3-YL) ETHYL]-N-(4-TERT-BUTYLPHENYL)FURAN-2-CARBOXAMIDE 0EN 3v3m 4.34 Specific

(2S)-2-({[(3S,4AR,8AS)-2-(BIPHENYL-4-YLCARBONYL) DECAHYDROISOQUINOLIN-3-YL]METHYL}AMINO)-3-(1H-IMIDAZOL-5-YL)PROPANAL 3BL 4twy 4.34 Specific

(4S,5Z)-4-AMINO-5-IMINOPENTANAMIDE 0JU 3vb4 4.34 Specific

N-[(5-METHYLISOXAZOL-3-YL)CARBONYL]ALANYL-L-VALYL-N~1~-((1R,2Z)-4-(BENZYLOXY)-4-OXO-1-{[(3R)-2-OXOPYRROLIDIN-3-YL]METHYL}BUT-2-ENYL)-L-LEUCINAMIDE 3IH 2hob 4.34 Specific

N-[(BENZYLOXY)CARBONYL]-O-TERT-BUTYL-L-SERYL-N-{(2R)-5-ETHOXY-5-OXO-1-[(3S)-2-OXOPYRROLIDIN-3-YL]PENTAN-2-YL}-L-PHENYLALANINAMIDE G85 3tnt 4.34 Specific

(4S,5Z)-4-AMINO-5-IMINOPENTANAMIDE 0JU 3vb6 4.34 Specific

N-[(BENZYLOXY)CARBONYL]-O-(TERT-BUTYL)-L-THREONYL-3-CYCLOHEXYL-N-[(1S)-2-HYDROXY-1-{[(3S)-2-OXOPYRROLIDIN-3-YL]METHYL}ETHYL]-L-ALANINAMIDE NOL 2gx4 4.34 Specific

N-[(BENZYLOXY)CARBONYL]-O-(TERT-BUTYL)-L-THREONYL-3-CYCLOHEXYL-N-[(1S)-2-HYDROXY-1-{[(3S)-2-OXOPYRROLIDIN-3-YL]METHYL}ETHYL]-L-ALANINAMIDE NOL 2gx4 4.34 Specific

(5S,8S,14R)-ETHYL 11-(3-AMINO-3-OXOPROPYL)-8-BENZYL-14-HYDROXY-5-ISOBUTYL-3,6,9,12-TETRAOXO-1-PHENYL-2-OXA-4, 7,10,11-TETRAAZAPENTADECAN-15-OATE AZP 2gtb 4.34 Specific

(5S,8S,14R)-ETHYL 11-(3-AMINO-3-OXOPROPYL)-8-BENZYL-14-HYDROXY-5-ISOBUTYL-3,6,9,12-TETRAOXO-1-PHENYL-2-OXA-4, 7,10,11-TETRAAZAPENTADECAN-15-OATE AZP 2a5i 4.34 Specific

(5S,8S,14R)-ETHYL 11-(3-AMINO-3-OXOPROPYL)-8-BENZYL-14-HYDROXY-5-ISOBUTYL-3,6,9,12-TETRAOXO-1-PHENYL-2-OXA-4, 7,10,11-TETRAAZAPENTADECAN-15-OATE AZP 2a5i 4.34 Specific

4-METHYLBENZENE-1,2-DITHIOL TLD 2z94 4.34 Specific

ETHYL (4R)-4-({N-[(BENZYLOXY)CARBONYL]-L-PHENYLALANYL}AMINO)-5-[(3S)-2-OXOPYRROLIDIN-3-YL] PENTANOATE G75 3szn 4.34 Specific

(5S,8S,14R)-ETHYL 11-(3-AMINO-3-OXOPROPYL)-8-BENZYL-14-HYDROXY-5-ISOBUTYL-3,6,9,12-TETRAOXO-1-PHENYL-2-OXA-4, 7,10,11-TETRAAZAPENTADECAN-15-OATE AZP 2a5k 4.34 Specific

(3S)-3-[(2S)-2-AMINO-3-HYDROXYBUTYL]PYRROLIDIN-2-ONE ECQ 2z3d 4.34 Specific

ETHYL (5S,8S,11R)-8-BENZYL-5-(2-TERT-BUTOXY-2-OXOETHYL)-3,6,9-TRIOXO-11-{[(3S)-2-OXOPYRROLIDIN-3-YL]METHYL}-1-PHENYL-2-OXA-4,7,10-TRIAZATETRADECAN-14-OATE G83 3tns 4.34 Specific

N-[(5-METHYLISOXAZOL-3-YL)CARBONYL]-L-ALANYL-L-VALYL-N~1~-((1S)-4-ETHOXY-4-OXO-1-{[(3S)-2-OXOPYRROLIDIN-3-YL]METHYL}BUT-2-ENYL)-L-LEUCINAMIDE I12 1wof 4.34 Specific

N-[(BENZYLOXY)CARBONYL]-O-TERT-BUTYL-L-THREONYL-N-[(1R)-4-CYCLOPROPYL-4-OXO-1-{[(3S)-2-OXOPYRROLIDIN-3-YL]METHYL}BUTYL]-L-LEUCINAMIDE ZU5 2zu5 4.34 Specific

N-[(BENZYLOXY)CARBONYL]-O-TERT-BUTYL-L-THREONYL-N-[(1R)-4-CYCLOPROPYL-4-OXO-1-{[(3S)-2-OXOPYRROLIDIN-3-YL]METHYL}BUTYL]-L-LEUCINAMIDE ZU5 2zu5 4.34 Specific

ETHYL (2E,4S)-4-[((2R)-2-{[N-(TERT-BUTOXYCARBONYL)-L-VALYL]AMINO}-2-PHENYLETHANOYL)AMINO]-5-[(3S)-2-OXOPYRROLIDIN-3-YL]PENT-2-ENOATE ENB 2d2d 4.34 Specific

ETHYL (5S,8S,11R)-8-BENZYL-5-(3-TERT-BUTOXY-3-OXOPROPYL)-3,6,9-TRIOXO-11-{[(3S)-2-OXOPYRROLIDIN-3-YL]METHYL}-1-PHENYL-2-OXA-4,7,10-TRIAZATETRADECAN-14-OATE G82 3tiu 4.34 Specific

DIAMINOZINC DAZ 2z9l 4.34 Specific

N-[(5-METHYLISOXAZOL-3-YL)CARBONYL]-L-ALANYL-L-VALYL-N~1~-((1S)-4-ETHOXY-4-OXO-1-{[(3S)-2-OXOPYRROLIDIN-3-YL]METHYL}BUT-2-ENYL)-L-LEUCINAMIDE I12 1wof 4.34 Specific

(4S,5Z)-4-AMINO-5-IMINOPENTANAMIDE 0JU 3vb5 4.34 Specific

(2S)-2-({[(3S,4AR,8AS)-2-(4-BROMOBENZOYL) DECAHYDROISOQUINOLIN-3-YL]METHYL}AMINO)-3-(1H-IMIDAZOL-5-YL)PROPANAL 3A7 4tww 4.34 Specific

NALPHA-[(BENZYLOXY)CARBONYL]-N-[(1R)-4-HYDROXY-1-METHYL-2-OXOBUTYL]-L-PHENYLALANINAMIDE WR1 2op9 4.34 Specific

2-[(2,4-DICHLORO-5-METHYLPHENYL)SULFONYL]-1,3-DINITRO-5-(TRIFLUOROMETHYL)BENZENE D3F 2gz7 4.34 Specific

DIMETHYL SULFOXIDE DMS 2z9k 4.34 Non-specific

ETHYL (2E,4S)-4-[((2R)-2-{[N-(TERT-BUTOXYCARBONYL)-L-VALYL]AMINO}-2-PHENYLETHANOYL)AMINO]-5-[(3S)-2-OXOPYRROLIDIN-3-YL]PENT-2-ENOATE ENB 2d2d 4.34 Specific

ETHYL (4R)-4-{[N-(TERT-BUTOXYCARBONYL)-L-PHENYLALANYL]AMINO}-5-[(3S)-2-OXOPYRROLIDIN-3-YL] PENTANOATE G81 3tit 4.34 Specific

(4S,5Z)-4-AMINO-5-IMINOPENTANAMIDE 0JU 3vb7 4.34 Specific

(3S)-3-[(2S)-2-AMINO-3-HYDROXYBUTYL]PYRROLIDIN-2-ONE ECQ 2z3c 4.34 Specific

N-[(2S)-1-HYDROXY-3-PHENYLPROPAN-2-YL]-NALPHA-[(2E)-3-PHENYLPROP-2-ENOYL]-L-PHENYLALANINAMIDE S89 3sn8 4.34 Specific

ACETYL GROUP ACE 2z3c 4.34 Non-specific

(3S)-3-[(2S)-2-AMINO-3-OXOBUTYL]PYRROLIDIN-2-ONE KCQ 2z3e 4.34 Specific

(3S)-3-[(2S)-2-AMINO-3-OXOBUTYL]PYRROLIDIN-2-ONE KCQ 2z3e 4.34 Specific

BENZYL (2-OXOPROPYL)CARBAMATE 959 3d62 4.34 Specific

N-(3-FUROYL)-D-VALYL-L-VALYL-N~1~-((1R,2Z)-4-ETHOXY-4-OXO-1-{[(3S)-2-OXOPYRROLIDIN-3-YL]METHYL}BUT-2-ENYL)-D-LEUCINAMIDE 9IN 2amd 4.34 Specific

N-((3S,6R)-6-((S,E)-4-ETHOXYCARBONYL-1-((S)-2-OXOPYRROLIDIN-3-YL)BUT-3-EN-2-YLCARBAMOYL)-2,9-DIMETHYL-4-OXODEC-8-EN-3-YL)-5-METHYLISOXAZOLE-3-CARBOXAMIDE CY6 2alv 4.34 Specific

(DIMETHYLAMINO)(HYDROXY)ZINC' DOZ 2z9k 4.34 Specific

(4S,5Z)-4-AMINO-5-IMINOPENTANAMIDE 0JU 3vb5 4.34 Specific

DIMETHYL SULFOXIDE DMS 2z9j 4.34 Non-specific

4-(DIMETHYLAMINO)BENZOIC ACID XP1 2v6n 4.34 Specific

DIMETHYL SULFOXIDE DMS 2z9l 4.34 Non-specific

ETHYL (4R)-4-{[(2R,5S)-5-{[N-(TERT-BUTOXYCARBONYL)-L-SERYL]AMINO}-6-METHYL-2-(3-METHYLBUT-2-EN-1-YL)-4-OXOHEPTANOYL]AMINO}-5-[(3R)-2-OXOPYRROLIDIN-3-YL]PENTANOATE CYV 2qiq 4.34 Specific

4-(DIMETHYLAMINO)BENZOIC ACID XP1 2vj1 4.34 Specific

BENZYL CHLOROCARBONATE PHQ 3vb5 4.34 Specific

ZINC(II)HYDROGENSULFIDE DTZ 2z9j 4.34 Specific

BENZYL CHLOROCARBONATE PHQ 3vb5 4.34 Specific

ACETYL GROUP ACE 3snb 4.34 Non-specific

DIMETHYL SULFOXIDE DMS 2z9k 4.34 Non-specific

(DIMETHYLAMINO)(HYDROXY)ZINC' DOZ 2z9k 4.34 Specific

(2S)-2-({[(3R,4AS,8AR)-2-(BIPHENYL-4-YLCARBONYL) DECAHYDROISOQUINOLIN-3-YL]METHYL}AMINO)-3-(1H-IMIDAZOL-5-YL)PROPANAL 3X5 4wy3 4.34 Specific

CHLOROACETONE ATO 1uk4 4.34 Specific

N-[(5-METHYLISOXAZOL-3-YL)CARBONYL]ALANYL-L-VALYL-N~1~-((1R,2Z)-4-(BENZYLOXY)-4-OXO-1-{[(3R)-2-OXOPYRROLIDIN-3-YL]METHYL}BUT-2-ENYL)-L-LEUCINAMIDE 3IH 2amq 4.34 Specific

DIMETHYL SULFOXIDE DMS 4mds 4.34 Non-specific

DIMETHYL SULFOXIDE DMS 3v3m 4.34 Non-specific

1,2-ETHANEDIOL EDO 3vb3 4.34 Non-specific

ETHYL (4R)-4-AMINO-5-[(3S)-2-OXOPYRROLIDIN-3-YL]PENTANOATE CEV 4rsp 3.93 Specific

N-{4-[(1H-BENZOTRIAZOL-1-YLACETYL)(THIOPHEN-3-YLMETHYL)AMINO]PHENYL}PROPANAMIDE R30 4ylu 3.93 Specific

TETRAETHYLENE GLYCOL PG4 4wmf 3.93 Specific

N-{4-[(1H-BENZOTRIAZOL-1-YLACETYL)(THIOPHEN-3-YLMETHYL)AMINO]PHENYL}PROPANAMIDE R30 4ylu 3.93 Specific

TRIETHYLENE GLYCOL PGE 4wmd 3.93 Non-specific

1,2-ETHANEDIOL EDO 4wme 3.93 Non-specific

N-{4-[(1H-BENZOTRIAZOL-1-YLACETYL)(THIOPHEN-3-YLMETHYL)AMINO]PHENYL}PROPANAMIDE R30 4ylu 3.93 Specific

N-{4-[(1H-BENZOTRIAZOL-1-YLACETYL)(THIOPHEN-3-YLMETHYL)AMINO]PHENYL}PROPANAMIDE R30 4ylu 3.93 Specific

TRIETHYLENE GLYCOL PGE 4wmd 3.93 Non-specific

ETHYL (4R)-4-AMINO-5-[(3S)-2-OXOPYRROLIDIN-3-YL]PENTANOATE CEV 4rsp 3.93 Specific

N-[(5-METHYLISOXAZOL-3-YL)CARBONYL]ALANYL-L-VALYL-N~1~-((1R,2Z)-4-(BENZYLOXY)-4-OXO-1-{[(3R)-2-OXOPYRROLIDIN-3-YL]METHYL}BUT-2-ENYL)-L-LEUCINAMIDE 3IH 3d23 3.72 Specific

N-[(5-METHYLISOXAZOL-3-YL)CARBONYL]ALANYL-L-VALYL-N~1~-((1R,2Z)-4-(BENZYLOXY)-4-OXO-1-{[(3R)-2-OXOPYRROLIDIN-3-YL]METHYL}BUT-2-ENYL)-L-LEUCINAMIDE 3IH 3d23 3.72 Specific

N-[(5-METHYLISOXAZOL-3-YL)CARBONYL]ALANYL-L-VALYL-N~1~-((1R,2Z)-4-(BENZYLOXY)-4-OXO-1-{[(3R)-2-OXOPYRROLIDIN-3-YL]METHYL}BUT-2-ENYL)-L-LEUCINAMIDE 3IH 3d23 3.72 Specific

N-[(5-METHYLISOXAZOL-3-YL)CARBONYL]ALANYL-L-VALYL-N~1~-((1R,2Z)-4-(BENZYLOXY)-4-OXO-1-{[(3R)-2-OXOPYRROLIDIN-3-YL]METHYL}BUT-2-ENYL)-L-LEUCINAMIDE 3IH 3d23 3.72 Specific

N-[(5-METHYLISOXAZOL-3-YL)CARBONYL]ALANYL-L-VALYL-N~1~-((1R,2Z)-4-(BENZYLOXY)-4-OXO-1-{[(3R)-2-OXOPYRROLIDIN-3-YL]METHYL}BUT-2-ENYL)-L-LEUCINAMIDE 3IH 3d23 3.72 Specific

N-[(5-METHYLISOXAZOL-3-YL)CARBONYL]ALANYL-L-VALYL-N~1~-((1R,2Z)-4-(BENZYLOXY)-4-OXO-1-{[(3R)-2-OXOPYRROLIDIN-3-YL]METHYL}BUT-2-ENYL)-L-LEUCINAMIDE 3IH 3d23 3.72 Specific

N-[(5-METHYLISOXAZOL-3-YL)CARBONYL]ALANYL-L-VALYL-N~1~-((1R,2Z)-4-(BENZYLOXY)-4-OXO-1-{[(3R)-2-OXOPYRROLIDIN-3-YL]METHYL}BUT-2-ENYL)-L-LEUCINAMIDE 3IH 3d23 3.72 Specific

N-[(5-METHYLISOXAZOL-3-YL)CARBONYL]ALANYL-L-VALYL-N~1~-((1R,2Z)-4-(BENZYLOXY)-4-OXO-1-{[(3R)-2-OXOPYRROLIDIN-3-YL]METHYL}BUT-2-ENYL)-L-LEUCINAMIDE 3IH 3d23 3.72 Specific

N-[(5-METHYLISOXAZOL-3-YL)CARBONYL]ALANYL-L-VALYL-N~1~-((1R,2Z)-4-(BENZYLOXY)-4-OXO-1-{[(3R)-2-OXOPYRROLIDIN-3-YL]METHYL}BUT-2-ENYL)-L-LEUCINAMIDE 3IH 2q6f 3.57 Specific

N-[(5-METHYLISOXAZOL-3-YL)CARBONYL]ALANYL-L-VALYL-N~1~-((1R,2Z)-4-(BENZYLOXY)-4-OXO-1-{[(3R)-2-OXOPYRROLIDIN-3-YL]METHYL}BUT-2-ENYL)-L-LEUCINAMIDE 3IH 2q6f 3.57 Specific

N-[(BENZYLOXY)CARBONYL]-O-TERT-BUTYL-L-SERYL-N-{(2R)-5-ETHOXY-5-OXO-1-[(3S)-2-OXOPYRROLIDIN-3-YL]PENTAN-2-YL}-L-PHENYLALANINAMIDE G85 2ynb 3.57 Specific

(4R)-2-METHYLPENTANE-2,4-DIOL MRD 1p9u 3.38 Non-specific

(1S,2S)-2-({N-[(BENZYLOXY)CARBONYL]-L-LEUCYL}AMINO)-1-HYDROXY-3-[(3S)-2-OXOPYRROLIDIN-3-YL]PROPANE-1-SULFONIC ACID K36 4f49 3.38 Specific

(4R)-2-METHYLPENTANE-2,4-DIOL MRD 1lvo 3.38 Non-specific

(4R)-2-METHYLPENTANE-2,4-DIOL MRD 1lvo 3.38 Non-specific

(4R)-2-METHYLPENTANE-2,4-DIOL MRD 1lvo 3.38 Non-specific

(4R)-2-METHYLPENTANE-2,4-DIOL MRD 1lvo 3.38 Non-specific

(1S,2S)-2-({N-[(BENZYLOXY)CARBONYL]-L-LEUCYL}AMINO)-1-HYDROXY-3-[(3S)-2-OXOPYRROLIDIN-3-YL]PROPANE-1-SULFONIC ACID K36 4f49 3.38 Specific

(4R)-2-METHYLPENTANE-2,4-DIOL MRD 1lvo 3.38 Non-specific

CHLOROMETHANE 0QE 1p9u 3.38 Specific

(4R)-2-METHYLPENTANE-2,4-DIOL MRD 1lvo 3.38 Non-specific

(4R)-2-METHYLPENTANE-2,4-DIOL MRD 1lvo 3.38 Non-specific

(1S,2S)-2-({N-[(BENZYLOXY)CARBONYL]-L-LEUCYL}AMINO)-1-HYDROXY-3-[(3S)-2-OXOPYRROLIDIN-3-YL]PROPANE-1-SULFONIC ACID K36 4f49 3.38 Specific

(4R)-2-METHYLPENTANE-2,4-DIOL MRD 1lvo 3.38 Non-specific

N-[(5-METHYLISOXAZOL-3-YL)CARBONYL]-L-ALANYL-L-VALYL-N~1~-((1S)-4-ETHOXY-4-OXO-1-{[(3S)-2-OXOPYRROLIDIN-3-YL]METHYL}BUT-2-ENYL)-L-LEUCINAMIDE I12 2amp 3.38 Specific

1,4-DIETHYLENE DIOXIDE DIO 1lvo 3.38 Non-specific

1,4-DIETHYLENE DIOXIDE DIO 1lvo 3.38 Non-specific

(4R)-2-METHYLPENTANE-2,4-DIOL MRD 1lvo 3.38 Non-specific

N-[(5-METHYLISOXAZOL-3-YL)CARBONYL]-L-ALANYL-L-VALYL-N~1~-((1S)-4-ETHOXY-4-OXO-1-{[(3S)-2-OXOPYRROLIDIN-3-YL]METHYL}BUT-2-ENYL)-L-LEUCINAMIDE I12 2amp 3.38 Specific

(4R)-2-METHYLPENTANE-2,4-DIOL MRD 1lvo 3.38 Non-specific

(4R)-2-METHYLPENTANE-2,4-DIOL MRD 1lvo 3.38 Non-specific

(4R)-2-METHYLPENTANE-2,4-DIOL MRD 1p9u 3.38 Non-specific

SULFATE ION SO4 1p9u 3.38 Non-specific

(4R)-2-METHYLPENTANE-2,4-DIOL MRD 1p9u 3.38 Non-specific

SULFATE ION SO4 1p9u 3.38 Non-specific

(1S,2S)-2-({N-[(BENZYLOXY)CARBONYL]-L-LEUCYL}AMINO)-1-HYDROXY-3-[(3S)-2-OXOPYRROLIDIN-3-YL]PROPANE-1-SULFONIC ACID K36 4f49 3.38 Specific

(4R)-2-METHYLPENTANE-2,4-DIOL MRD 1lvo 3.38 Non-specific

(4R)-2-METHYLPENTANE-2,4-DIOL MRD 1p9u 3.38 Non-specific

CHLOROMETHANE 0QE 1p9u 3.38 Specific

(4S)-2-METHYL-2,4-PENTANEDIOL MPD 2zu2 2.96 Non-specific

1,4-DIETHYLENE DIOXIDE DIO 1p9s 2.96 Non-specific

With the binding site defined, we selected all superimposed ligands from the previous step for the calculation of the available docking volume. The receptor structure was generated using N3-3CL^{pro} crystal complex (PDB entry: 6LU7) with MakeReceptor GUI of OEDocking 3.2.0.2 software package (OpenEye Scientific Software, Inc., Santa Fe, NM, USA; www.eyesopen.com). A box with the volume of 18347 Å^3 (26.00 × 29.00 × 24.33 Å) was defined around all superimposed small-molecule ligands identified by ProBiS.[4] A balanced site shape potential was calculated where docking volume was 6799

Å^3 with applied central inner volume of 113 Å^3 for pose restriction. No constraints were used and active-site residue conformations or protonation states were maintained (Figure S1).

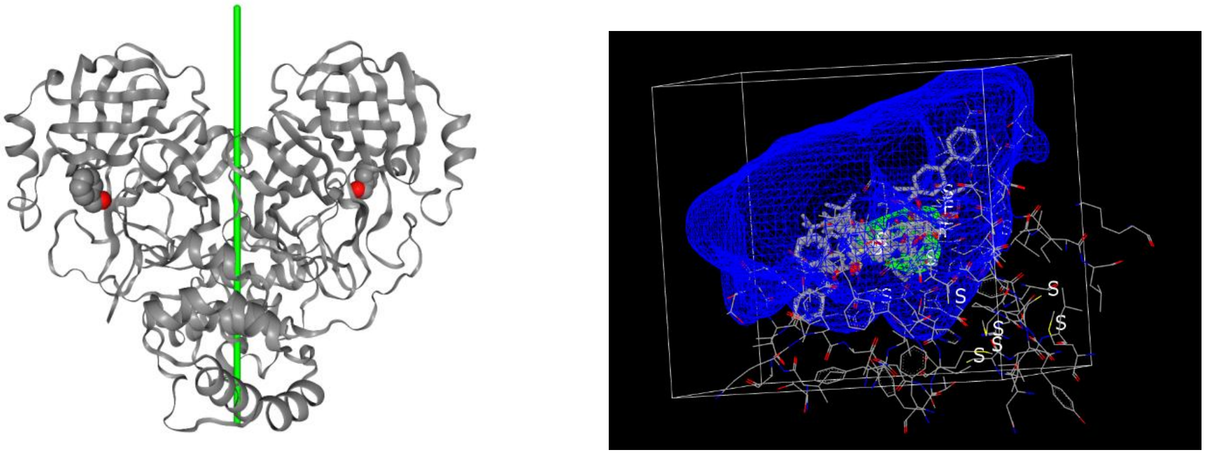

Figure S1: Left: PDB ID: 6LU7 crystal structure of the COVID-19 main protease C2 global symmetry. Two asymmetric units are depicted in gray-colored cartoon model with two active sites emphasized by gray-red space filled ligand moieties. Symmetry axis is presented in green; Right: The defined docking volume on the generated SARS-CoV-2 N3-3CL^{pro} crystal complex "receptor". The blue mesh depicts the docking volume while the central green mesh denotes the "inner contour". Molecular docking using the inner contour and OpenEye Fred software results in a rejection of any pose examined by the exhaustive search that does not touch this shape. Touching is obtained if the centre of at least one heavy atom from the examined ligand falls within this shape.

3. VIRTUAL SCREENING

VS was conducted using Fred software from OpenEye (OpenEye Scientific Software, Inc., Santa Fe, NM, USA; www.eyesopen.com). In the protocol normal resolution docking parameter and Chemgauss4 scoring function were used. [7-9] VS or HTVS was performed on Intel ® Xeon® E5-2630 v4 CPU and 32 GB of memory machines using Linux operating system. The obtained results as tops scoring hits are gathered in Table S2. As a proof-of-concept experiment, crystal structure (PDB ID: 6LU7) native ligand N3 covalent bond with Cys145 was cleaved, target prepared and noncovalent ligand re-docking performed with a docking score of -5.1.

molecules

4. MD CLUSTERING

Theseus clustering [11]

< BEGIN THESEUS 1.3.5 >

I===-===-===-===-===-===-===-===-===-===-===-===-===-===-===-===-===-===-===-=I

I THESEUS: Maximum likelihood multiple superpositioning I

I=-===-===-===-===-===-===-===-===-===-===-===-===-===-===-===-===-===-===-===I

Reading pdb file ...

Successfully read 101 models and/or structures

Selecting coordinates for superposition ...

Calculating superposition transformations ...

Calculating statistics ...

Calculating likelihood statistics ...

101 models superimposed in 4780.0 ms

* Least-squares <sigma> 0.74558

* Classical LS pairwise <RMSD> 1.83541

* Maximum Likelihood <sigma> 0.45521

~ Log Likelihood -1210087.41

~ AIC -1229734.17

~ BIC -1347363.98

+ Rotational, translational, covar chi^2 13.66 (P:0.00e+00)

+ Hierarchical minimum var (sigma) 6.77e-04 (2.60e-02)

+ Hierarchical var (1.04e-01, 5.00e-01) chi^2 17.38 (P:0.00e+00)

+ Omnibus chi^2 13.66 (P:0.00e+00)

< skewness 0.00 (P:1.94e-01)

< skewness Z-value 1.30

< kurtosis 0.60 (P:0.00e+00)

< kurtosis Z-value 145.09

FP error in transformed coordinates: 3.52e-15

Minimum RMSD error per atom: 1.33e-06

Data pts = 1421979, Free params = 19379, D/P = 73.4

* Median structure = #57

N(total) = 473993, N(atoms) = 4693, N(structures) = 101

Total rounds = 17

Converged to a fractional precision of 7.8e-08

I===-===-===-===-===-===-===-===-===-===-===-===-===-===-===-===-===-===-===-==I

Transforming coordinates ...

Writing transformed coordinates PDB file ...

Writing average coordinate file ...

Done.

I===-===-===-===-===-===-===-===-===-===-===-===-===-===-===-===-===-===-===-==I

< END THESEUS 1.3.5 >

MD trajectory clustering with ClusCo [10]

Score: rmsd

Filename: filelist.txt (conformers: 101)

Hierarchical clustering, pairwise average-linkage, K=7

0 : model_45.pdb : 0.386 : 1.1 : model_1.pdb model_4.pdb model_6.pdb model_7.pdb model_9.pdb model_11.pdb model_12.pdb model_13.pdb model_14.pdb model_17.pdb model_18.pdb model_20.pdb model_21.pdb model_22.pdb model_23.pdb model_25.pdb model_26.pdb model_27.pdb model_29.pdb model_30.pdb model_34.pdb model_37.pdb model_38.pdb model_41.pdb model_42.pdb model_43.pdb model_44.pdb model_45.pdb model_46.pdb model_47.pdb model_48.pdb model_49.pdb model_50.pdb model_51.pdb model_52.pdb model_53.pdb model_54.pdb model_66.pdb model_70.pdb

1 : model_86.pdb : 0.317 : 1.1 : model_55.pdb model_56.pdb model_64.pdb model_65.pdb model_67.pdb model_68.pdb model_69.pdb model_71.pdb model_72.pdb model_74.pdb model_75.pdb model_76.pdb model_77.pdb model_79.pdb model_80.pdb model_81.pdb model_82.pdb model_83.pdb model_84.pdb model_85.pdb model_86.pdb model_88.pdb model_89.pdb model_91.pdb model_92.pdb model_93.pdb model_95.pdb model_96.pdb model_97.pdb model_98.pdb model_99.pdb model_100.pdb

2 : model_40.pdb : 0.099 : 1.1 : model_5.pdb model_15.pdb model_19.pdb model_24.pdb model_28.pdb model_31.pdb model_32.pdb model_33.pdb model_39.pdb model_40.pdb

3 : model_62.pdb : 0.0693 : 1.1 : model_35.pdb model_58.pdb model_59.pdb model_60.pdb model_61.pdb model_62.pdb model_63.pdb

4 : model_8.pdb : 0.0594 : 1.1 : model_2.pdb model_3.pdb model_8.pdb model_10.pdb model_16.pdb model_36.pdb

5 : model_94.pdb : 0.0495 : 1 : model_57.pdb model_78.pdb model_87.pdb model_94.pdb model_101.pdb

6 : model_73.pdb : 0.0198 : 1.1 : model_73.pdb model_90.pdb

Score: rmsd

molecules MDPI

Filename: filelist.txt (conformers: 101)

Hierarchical clustering, pairwise average-linkage, K=7

model_1.pdb	2
model_2.pdb	3
model_3.pdb	3
model_4.pdb	2
model_5.pdb	1
model_6.pdb	2
model_7.pdb	2
model_8.pdb	3 *
model_9.pdb	2
model_10.pdb	3
model_11.pdb	2
model_12.pdb	2
model_13.pdb	2
model_14.pdb	2
model_15.pdb	1
model_16.pdb	3
model_17.pdb	2
model_18.pdb	2
model_19.pdb	1
model_20.pdb	2

model_21.pdb	2
model_22.pdb	2
model_23.pdb	2
model_24.pdb	1
model_25.pdb	2
model_26.pdb	2
model_27.pdb	2
model_28.pdb	1
model_29.pdb	2
model_30.pdb	2
model_31.pdb	1
model_32.pdb	1
model_33.pdb	1
model_34.pdb	2
model_35.pdb	0
model_36.pdb	3
model_37.pdb	2
model_38.pdb	2
model_39.pdb	1
model_40.pdb	1 *
model_41.pdb	2
model_42.pdb	2
model_43.pdb	2

model_44.pdb	2
model_45.pdb	2 *
model_46.pdb	2
model_47.pdb	2
model_48.pdb	2
model_49.pdb	2
model_50.pdb	2
model_51.pdb	2
model_52.pdb	2
model_53.pdb	2
model_54.pdb	2
model_55.pdb	4
model_56.pdb	4
model_57.pdb	5
model_58.pdb	0
model_59.pdb	0
model_60.pdb	0
model_61.pdb	0
model_62.pdb	0 *
model_63.pdb	0
model_64.pdb	4
model_65.pdb	4
model_66.pdb	2

model_67.pdb	4
model_68.pdb	4
model_69.pdb	4
model_70.pdb	2
model_71.pdb	4
model_72.pdb	4
model_73.pdb	6 *
model_74.pdb	4
model_75.pdb	4
model_76.pdb	4
model_77.pdb	4
model_78.pdb	5
model_79.pdb	4
model_80.pdb	4
model_81.pdb	4
model_82.pdb	4
model_83.pdb	4
model_84.pdb	4
model_85.pdb	4
model_86.pdb	4 *
model_87.pdb	5
model_88.pdb	4
model_89.pdb	4

molecules MDPI

model_90.pdb	6
model_91.pdb	4
model_92.pdb	4
model_93.pdb	4
model_94.pdb	5 *
model_95.pdb	4
model_96.pdb	4
model_97.pdb	4
model_98.pdb	4
model_99.pdb	4
model_100.pdb	4
model_101.pdb	5

molecules

MDPI

5. LIGAND – RECEPTOR CONTACT ANALYSIS

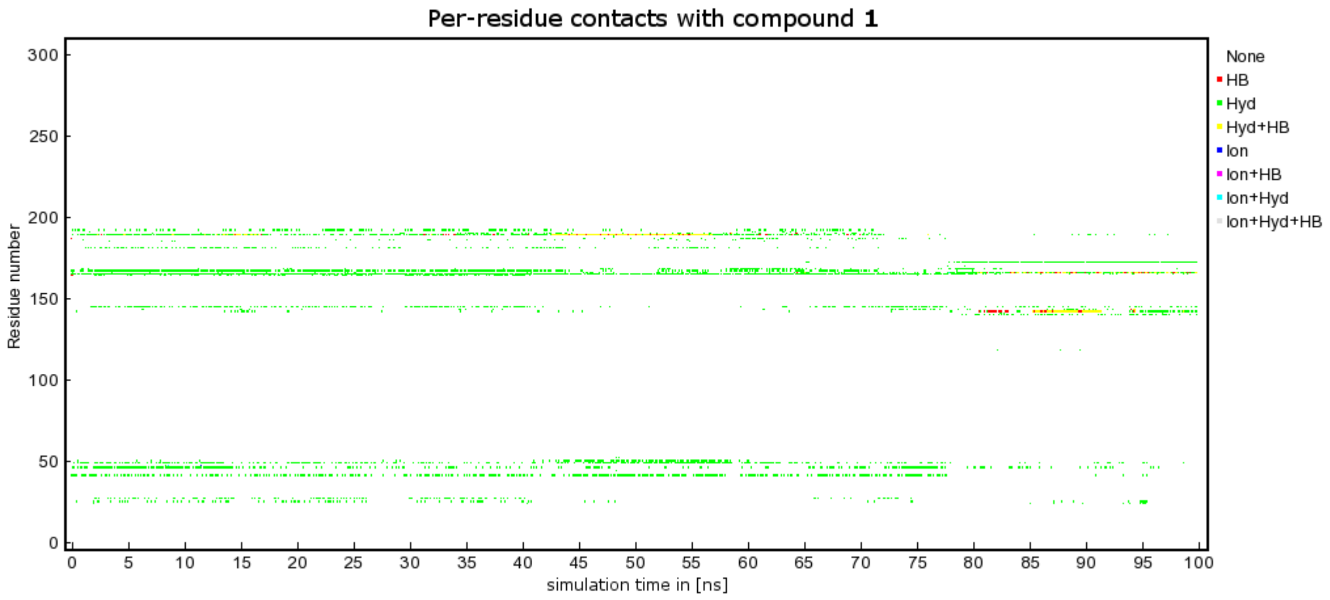

Figure S2: Compound **1** MD run 1.

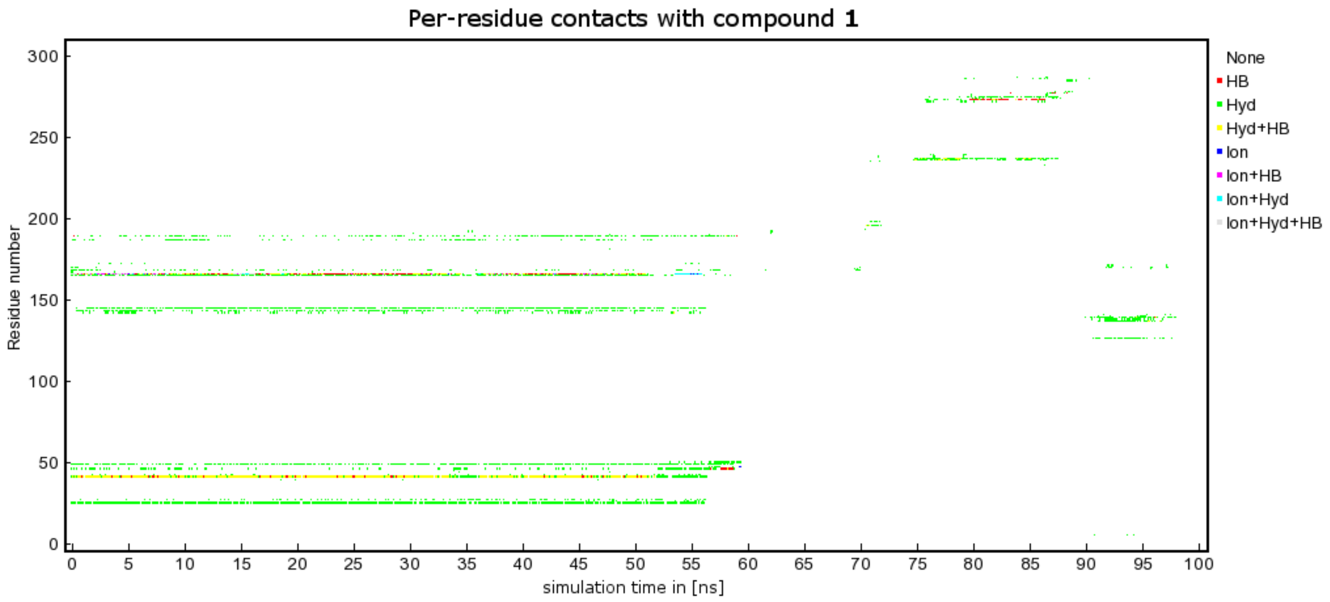

Figure S3: Compound **1** MD run 2.

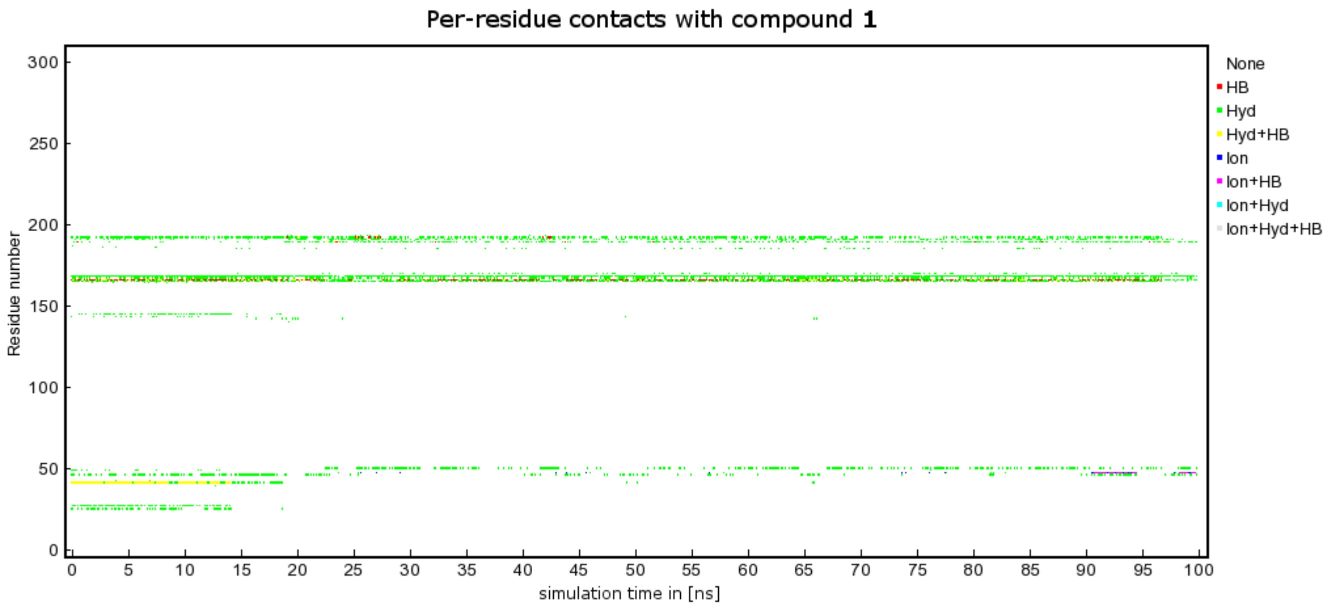

Figure S4: Compound **1** MD run 3.

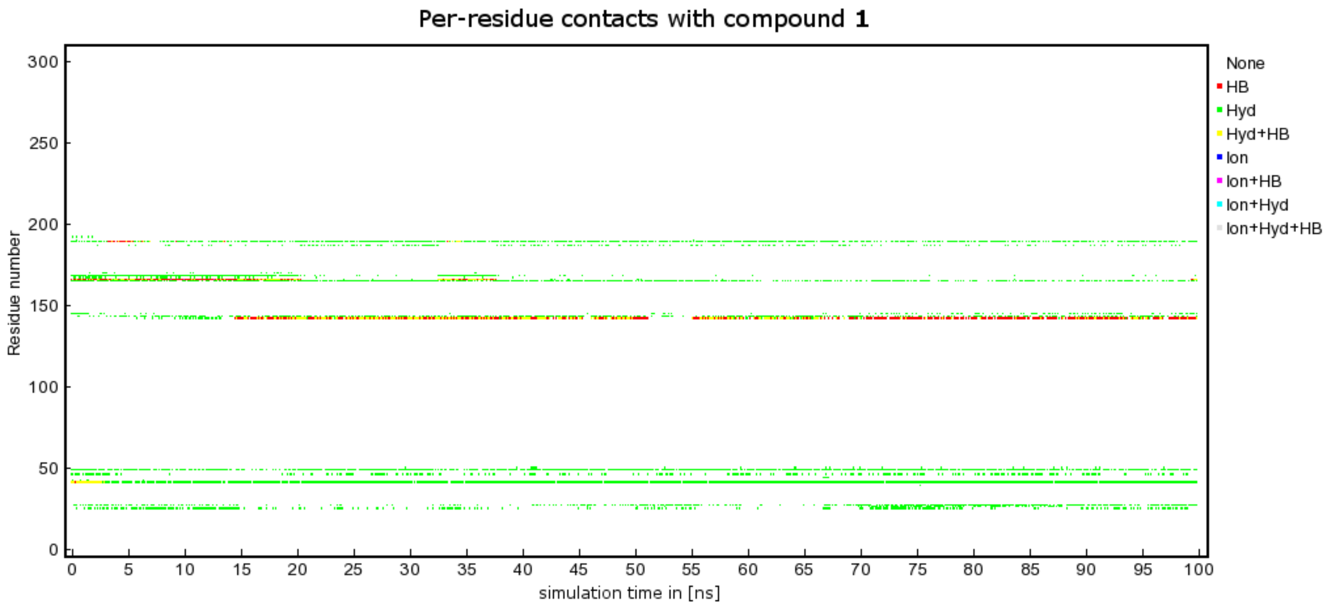

Figure S5: Compound **1** MD run 4.

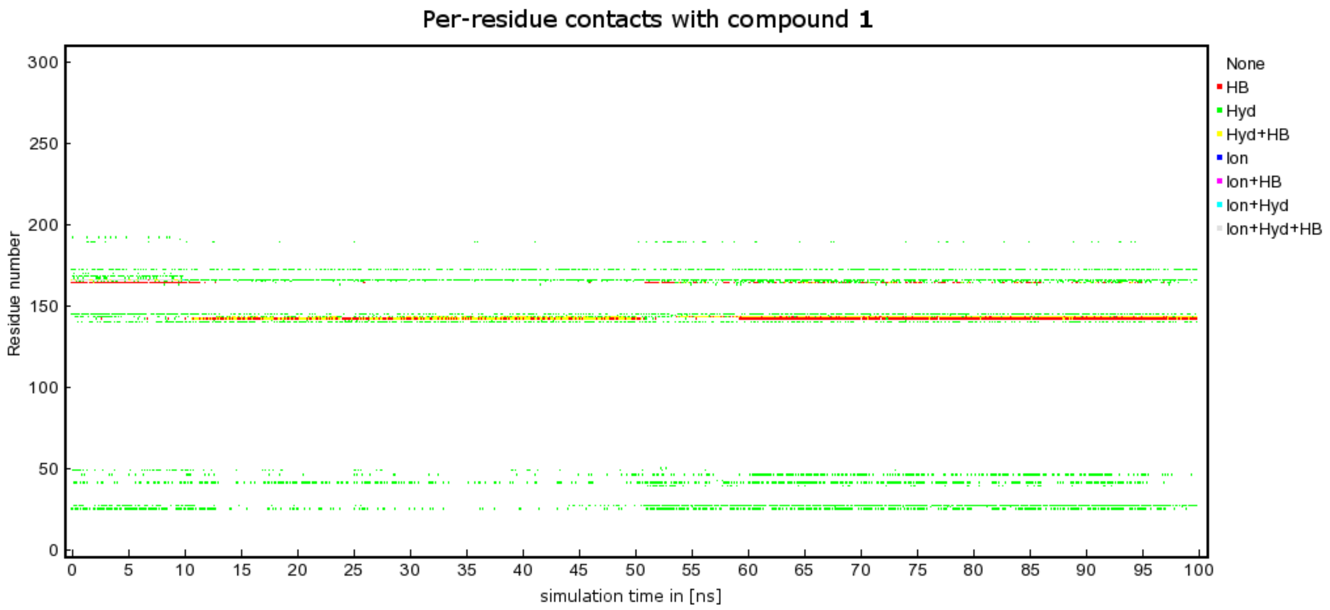

Figure S6: Compound **1** MD run 5.

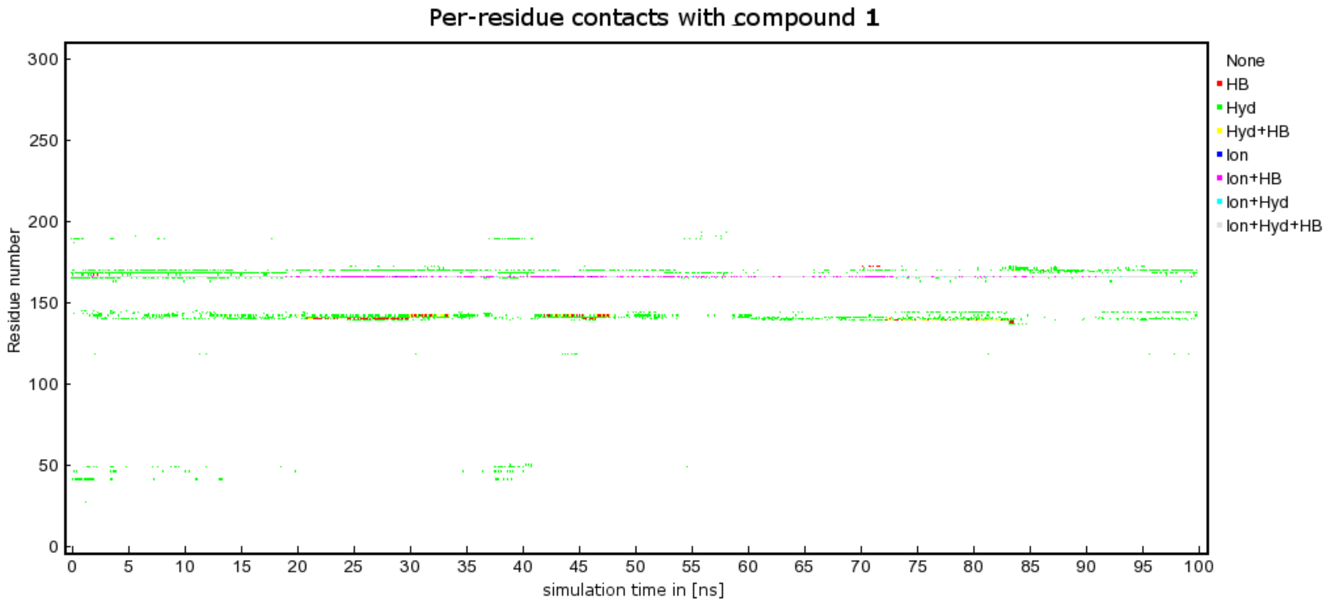

Figure S7: Compound **1** MD run 6.

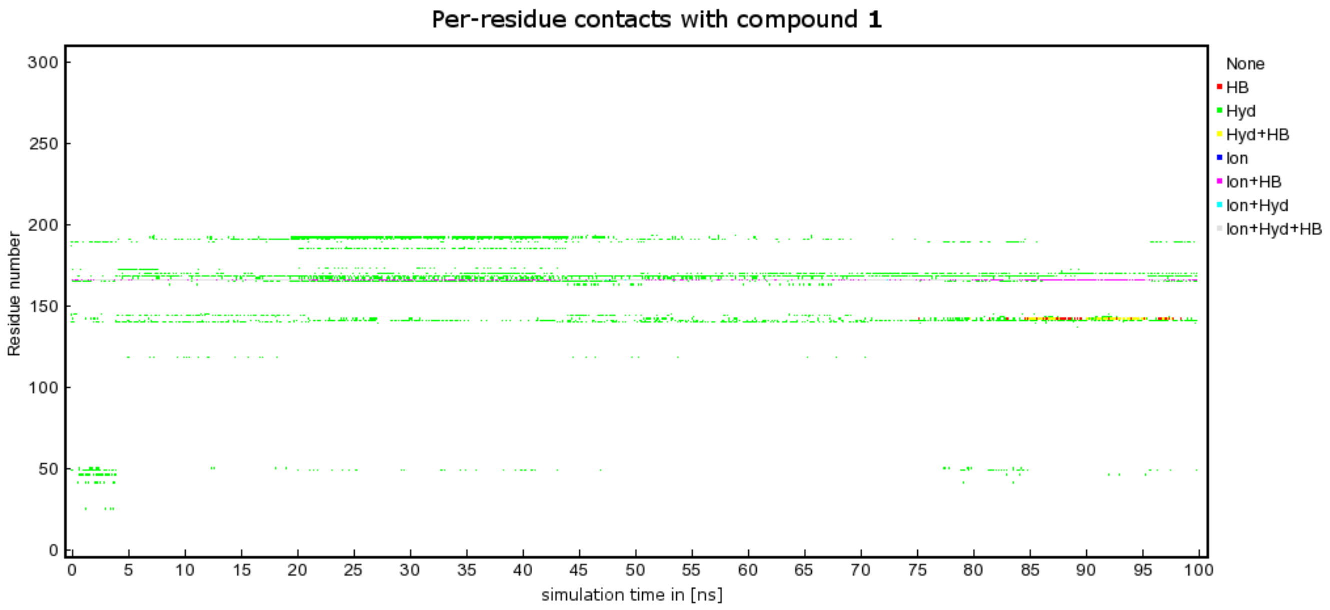

Figure S8: Compound **1** MD run 7.

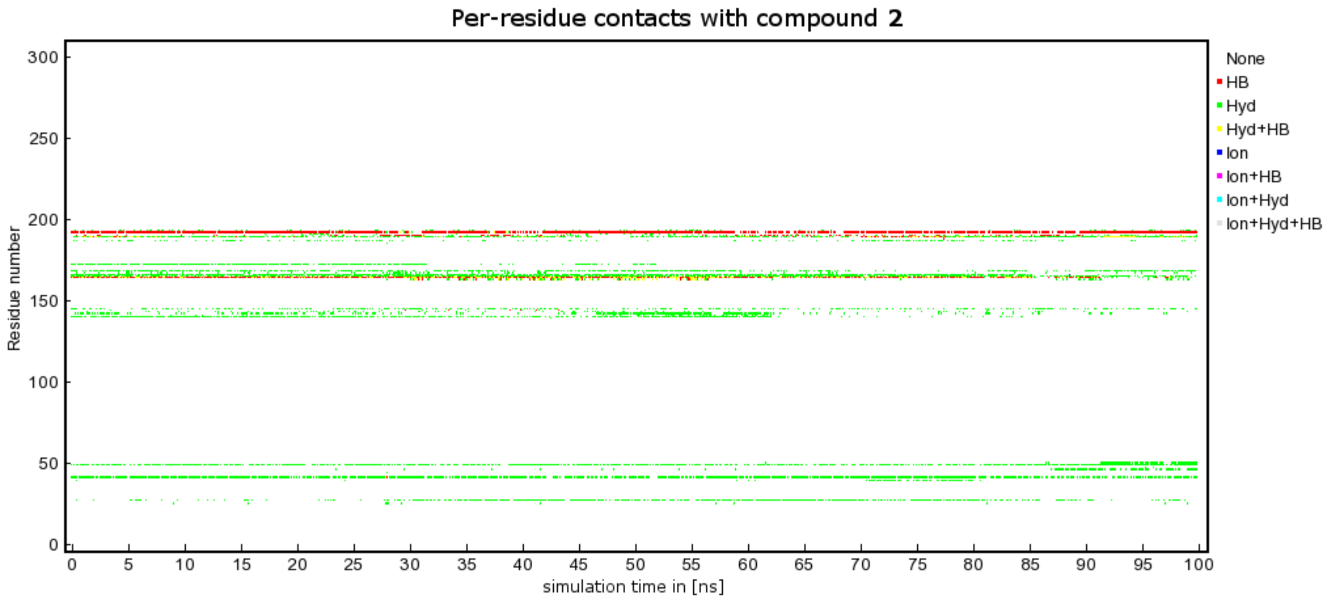

Figure S9: Compound **2** MD run 1.

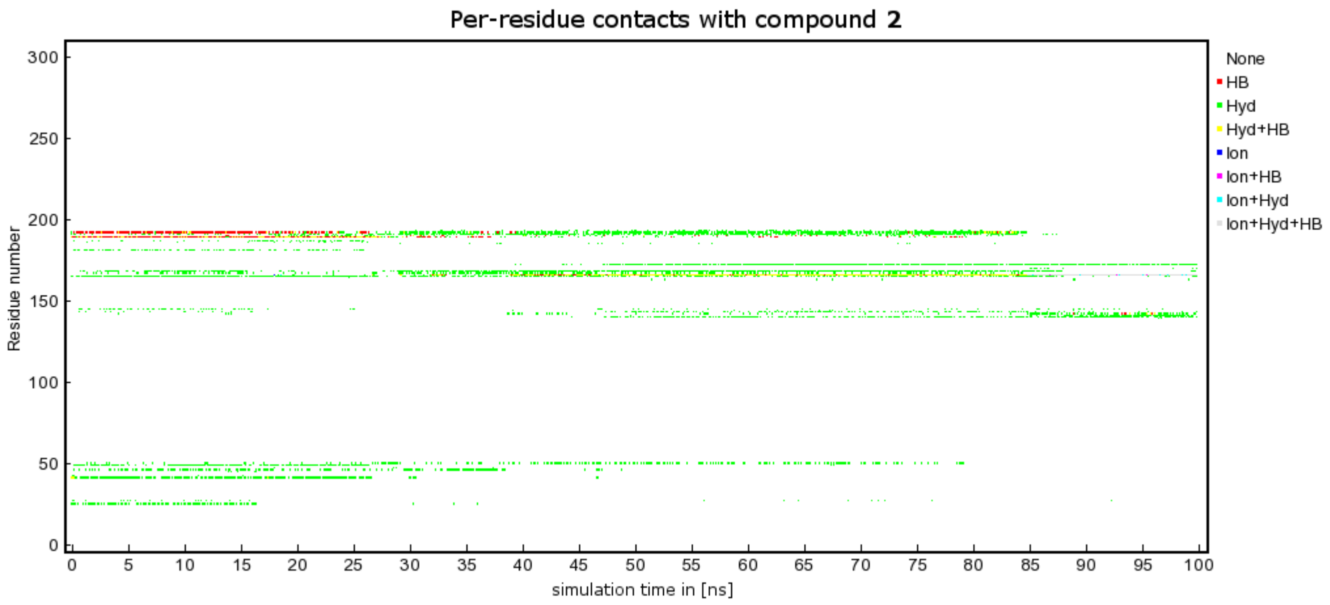

Figure S10: Compound **2** MD run 2.

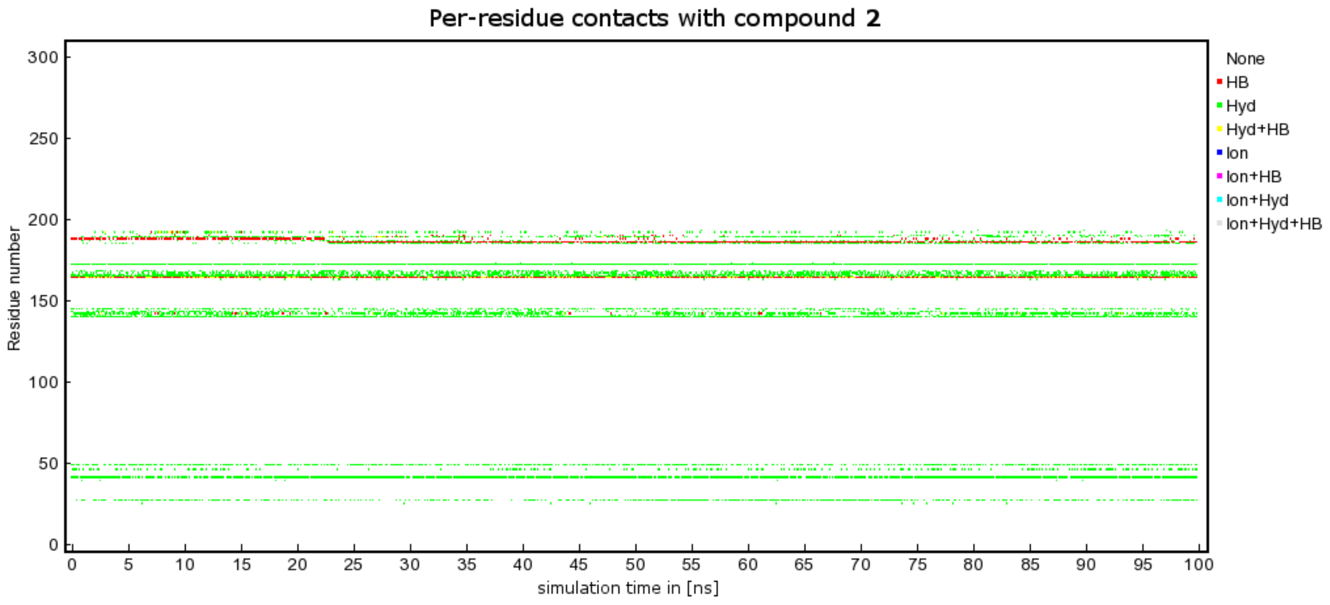

Figure S11: Compound **2** MD run 3.

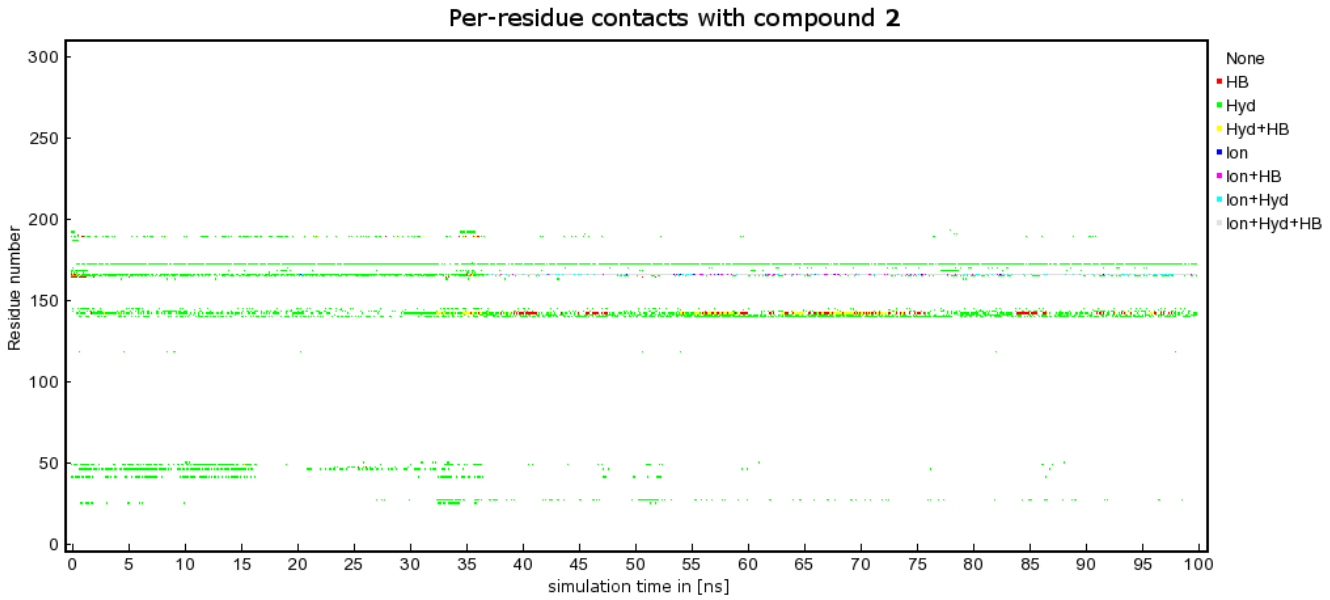

Figure S12: Compound **2** MD run 4.

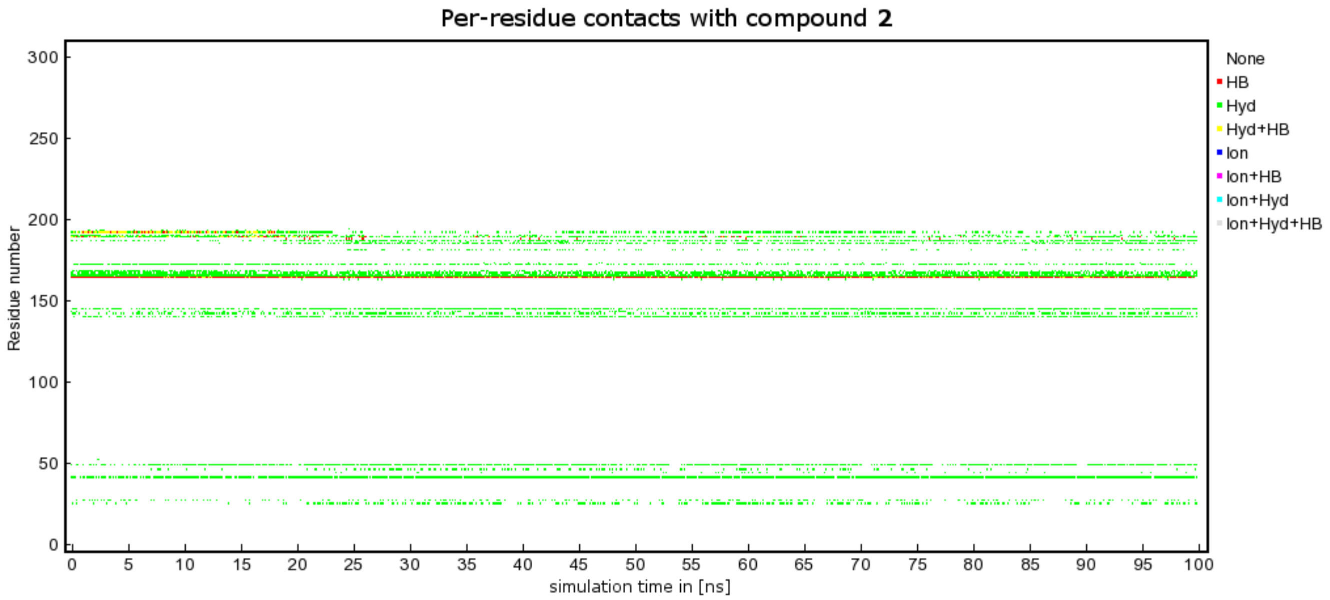

Figure S13: Compound **2** MD run 5.

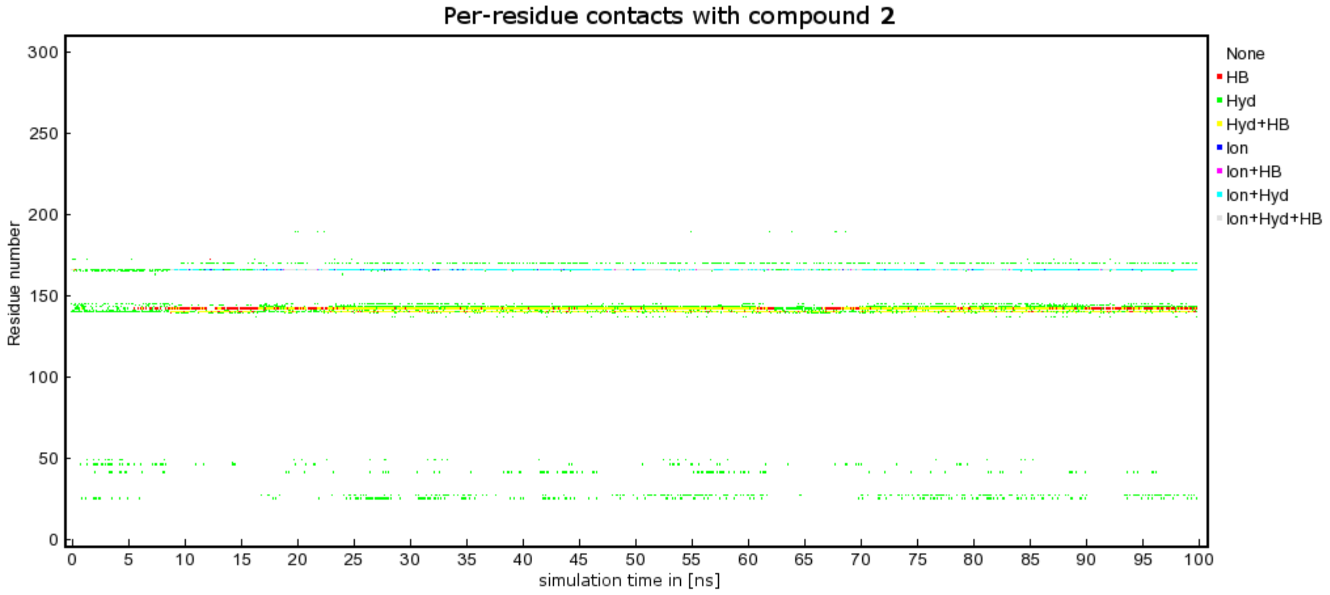

Figure S14: Compound **2** MD run 6.

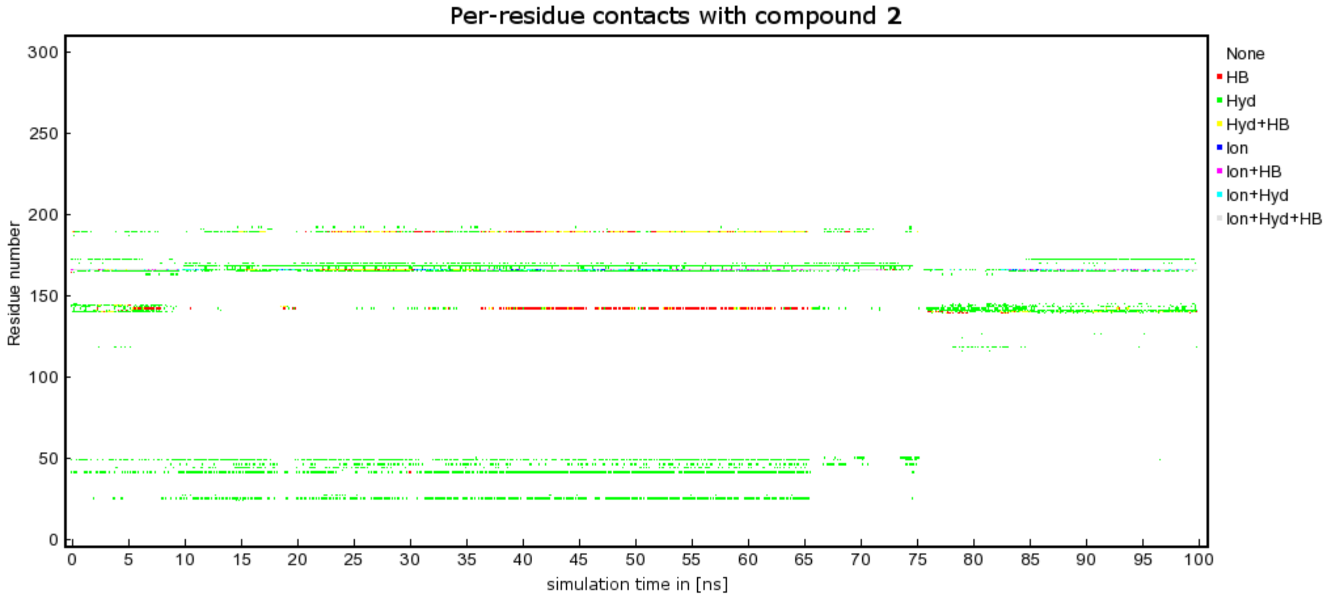

Figure S15: Compound **2** MD run 7.

6. BINDING MODE ANALYSIS (Figure S16, S17)

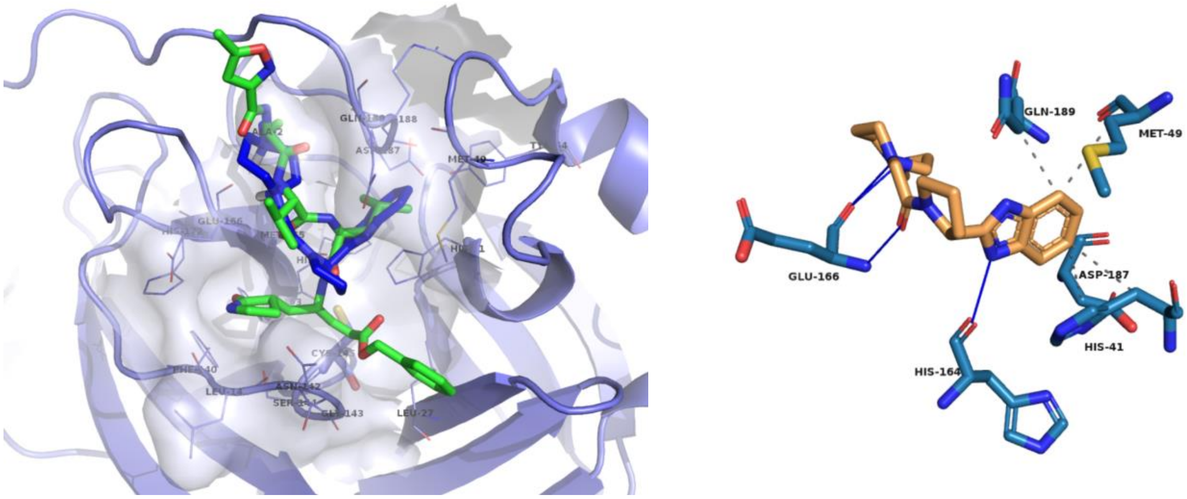

Figure S16: Left: PDB ID: 6LU7 crystal structure of the COVID-19 main protease with N3 crystalized inhibitor depicted in stick model colored green and compound **1** in blue colored stick model representation. Emphasized is catalytic Cys145 in stick model. Right: Emphasized contacts of compound **1** with its target SARS-CoV-2 $3CL^{pro}$.

Table S1: Hydrophobic interactions of Compound **1** with its target.

Index	Residue	AA	Distance	Ligand Atom	Protein Atom
1	41A	HIS	3.89	2392	307
2	49A	MET	3.57	2388	368
3	187A	ASP	3.80	2392	1442
4	189A	GLN	3.32	2388	1462

molecules

Table S2: Hydrogen bonds of Compound **1** with its target.

Index	Residue	AA	Distance H-A	Distance D-A	Donor Angle	Protein donor?	Sidechain	Donor Atom	Acceptor Atom
1	164A	HIS	1.87	2.83	157.44	✖	✖	2381 [Npl]	1266 [O2]
2	166A	GLU	1.75	2.72	156.68	✖	✖	2386 [N3+]	1284 [O2]
3	166A	GLU	1.82	2.75	155.52	✔	✖	1281 [Nam]	2368 [O2]
4	166A	GLU	2.16	3.12	153.15	✖	✖	2374 [N3+]	1284 [O2]

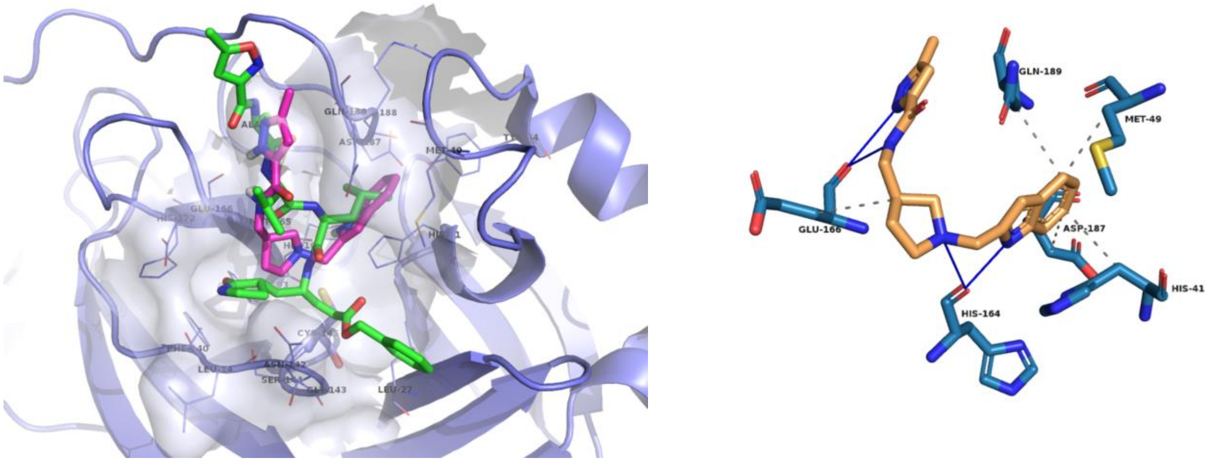

Figure S17: Left: PDB ID: 6LU7 crystal structure of the COVID-19 main protease with N3 crystalized inhibitor depicted in stick model colored green and compound **2** in blue colored stick model representation. Emphasized is catalytic Cys145 in stick model. Right: Emphasized contacts of compound **2** with its target SARS-CoV-2 3CL^{pro}.

molecules

MDPI

Table S3: Hydrophobic interactions of Compound **2** with its target.

Index	Residue	AA	Distance	Ligand Atom	Protein Atom
1	41A	HIS	3.63	24	356
2	49A	MET	3.41	23	417
3	166A	GLU	3.86	8	1334
4	187A	ASP	3.78	24	1491
5	189A	GLN	3.62	23	1511

Table S4: Hydrogen bonds of Compound **2** with its target.

Index	Residue	AA	Distance H-A	Distance D-A	Donor Angle	Protein donor?	Sidechain	Donor Atom	Acceptor Atom
1	164A	HIS	1.77	2.74	159.23	✖	✖	18 [Nar]	1315 [O2]
2	164A	HIS	2.61	3.48	141.55	✖	✖	14 [N3+]	1315 [O2]
3	166A	GLU	2.30	3.31	172.76	✖	✖	6 [Nar]	1333 [O2]
4	166A	GLU	2.43	3.45	173.81	✖	✖	3 [Nam]	1333 [O2]

Ensemble conformations:

molecules MDPI

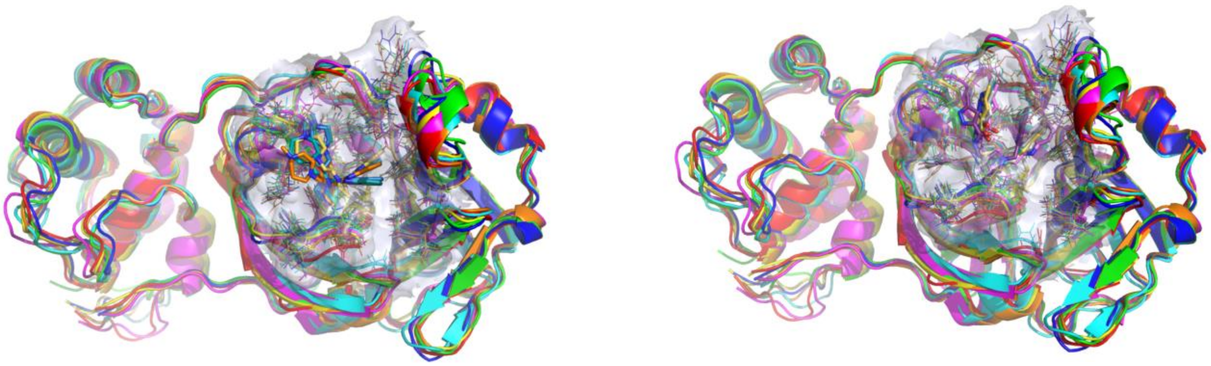

Figure S18: Left: MD ensemble of the SARS-CoV-2 main protease with compound **1** indicating protein flexibility. Right: MD ensemble of the PDB ID: 6LU7 with compound **2** and analogous ligand positioning.

7. *IN SILICO* HITS

Table S5: Top hits from the performed virtual screening (VS) or high throughput virtual screening (HTVS) on the SARS-CoV-2 main protease

no.	***structure***	***Mr (g/mol)***	***SMILES***	***Fred docking score***
1	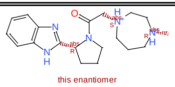	343.5	O=C(N3(C(C1(=NC=2(C=CC=CC=2(N1))))CCC3))C[NH+]4(CC[NH+](C)CCC4)	-14.5
2	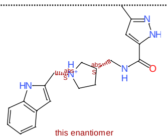	338.4	O=C(NCC3(C[NH+](CC=1(NC=2(C=CC=CC=2(C=1))))CC3))C=4(NN=C(C=4)C)	-13.5
3	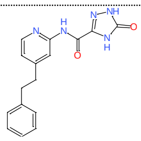	309.3	O=C1(NN=C(N1)C(=O)NC2(=NC=CC(=C2)CCC3(=CC=CC=C3)))	-13.0
4	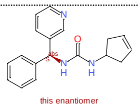	293.4	O=C(NC1(CC=CC1))NC(C2(=CN=CC=C2))C3(=CC=CC=C3)	-13.0
5	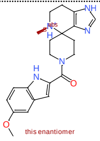	380.5	O=C(N3(CCC1([NH+](CCC2(=C1N=CN2))C)(CC3)))C=4(NC=5(C=CC(=CC=5(C=4))OC))	-13.0
6	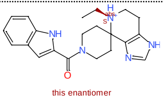	364.5	O=C(N3(CCC1([NH+](CCC2(=C1N=CN2))CC)(CC3)))C=4(NC=5(C=CC=CC=5(C=4)))	-13.0
7	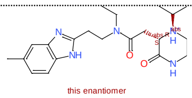	386.5	O=C1(NCC[NH+](C1CC(=O)N(CCC2(=NC=3(C=C(C)C=CC=3(N2))))CC)C(C)C)	-12.9
8	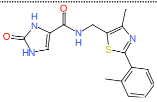	328.4	S1(C(=NC(=C1CNC(=O)C=2(NC(=O)NC=2))C)C3(=C(C=CC=C3)C))	-12.824043
9	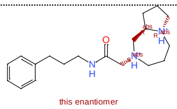	317.5	O=C(NCCCC1(=CC=CC=C1))C[NH+]3(CC2([NH+](CCC2)CCC3))	-12.8
10	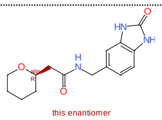	289.3	O=C2(NC=3(C=C(CNC(=O)CC1(OCCCC1))C=CC=3(N2)))	-12.8

11	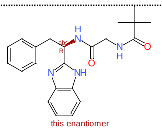	378.5	O=C(NCC(=O)NC(C1(=NC=2(C=CC=CC=2(N1))))CC3(=CC=CC=C3))C(C)(C)C	-12.7
12	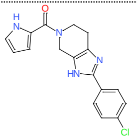	326.8	ClC4(=CC=C(C1(=NC=2(CCN(CC=2(N1))C(=O)C=3(NC=CC=3))))C=C4)	-12.5
13	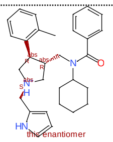	456.7	O=C(N(C1(CCCCC1))CC3(C(C2(=C(C=CC=C2)C))C[NH+](C3)CC=4(NC=CC=4)))C5(=CC=CC=C5)	-12.5
14	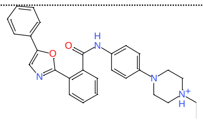	453.6	O=C(NC2(=CC=C(N1(CC[NH+](CC)CC1))C=C2))C5(=C(C=4(OC(C3(=CC=CC=C3))=CN=4))C=CC=C5)	-12.5
15	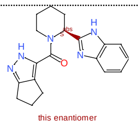	335.4	O=C(N3(C(C1(=NC=2(C=CC=CC=2(N1))))CCCC3))C=4(NN=C5(C=4CCC5))	-12.5
16	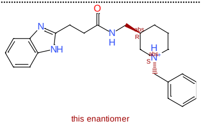	377.5	O=C(NCC1(C[NH+](CCC1)CC2(=CC=CC=C2)))CCC3(=NC=4(C=CC=CC=4(N3)))	-12.4
17	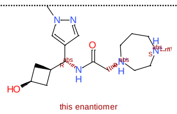	337.5	O=C(NC(C=1(C=NN(C=1)C))C2(CC(O)C2))C[NH+]3(CC[NH+](C)CCC3)	-12.4
18	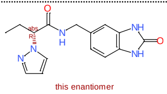	299.3	O=C2(NC=3(C=C(CNC(=O)C(N1(N=CC=C1))CC)C=CC=3(N2)))	-12.4
19	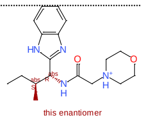	331.4	O=C(NC(C1(=NC=2(C=CC=CC=2(N1))))C(CC)C)C[NH+]3(CCOCC3)	-12.4
20	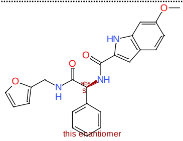	403.4	O=C(NC(C1(=CC=CC=C1))C(=O)NCC=2(OC=CC=2))C=3(NC=4(C=C(OC)C=CC=4(C=3)))	-12.3

As an experiment, crystal structure (PDB ID: 6LU7) native ligand N3 covalent bond towards Cys145 was cleaved, compound prepared and noncovalent ligand re-docking was performed with FRED docking score of -5.1.

8. Top-Scoring library plots

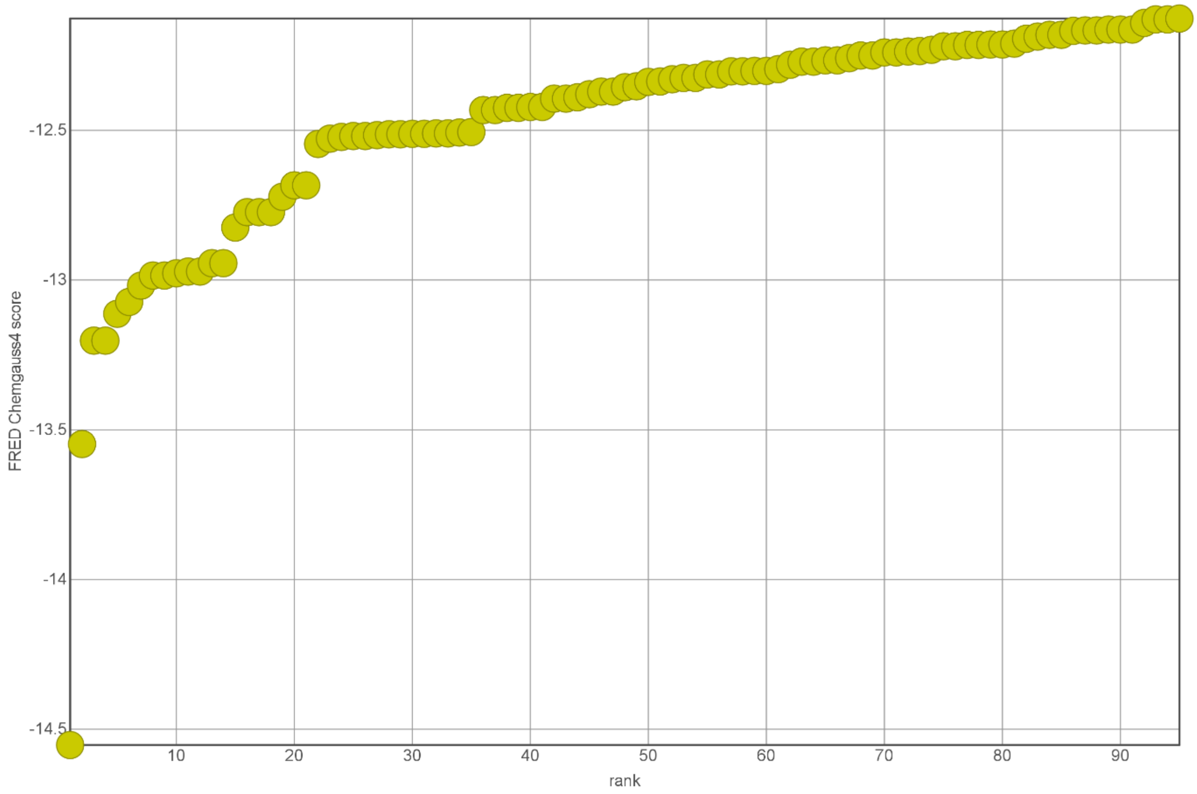

Figure S19: Compound rank vs FRED Chemgauss4 score.

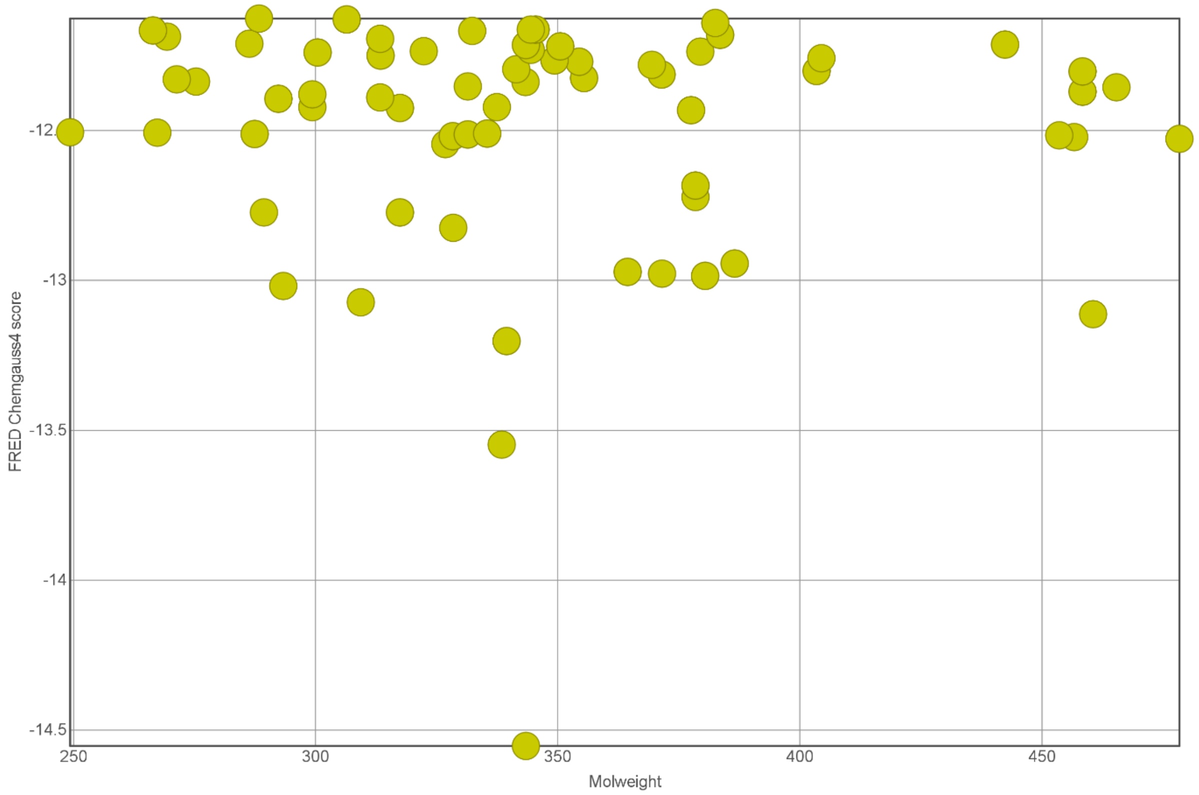

Figure S20: Compound MW vs FRED Chemgauss4 score.

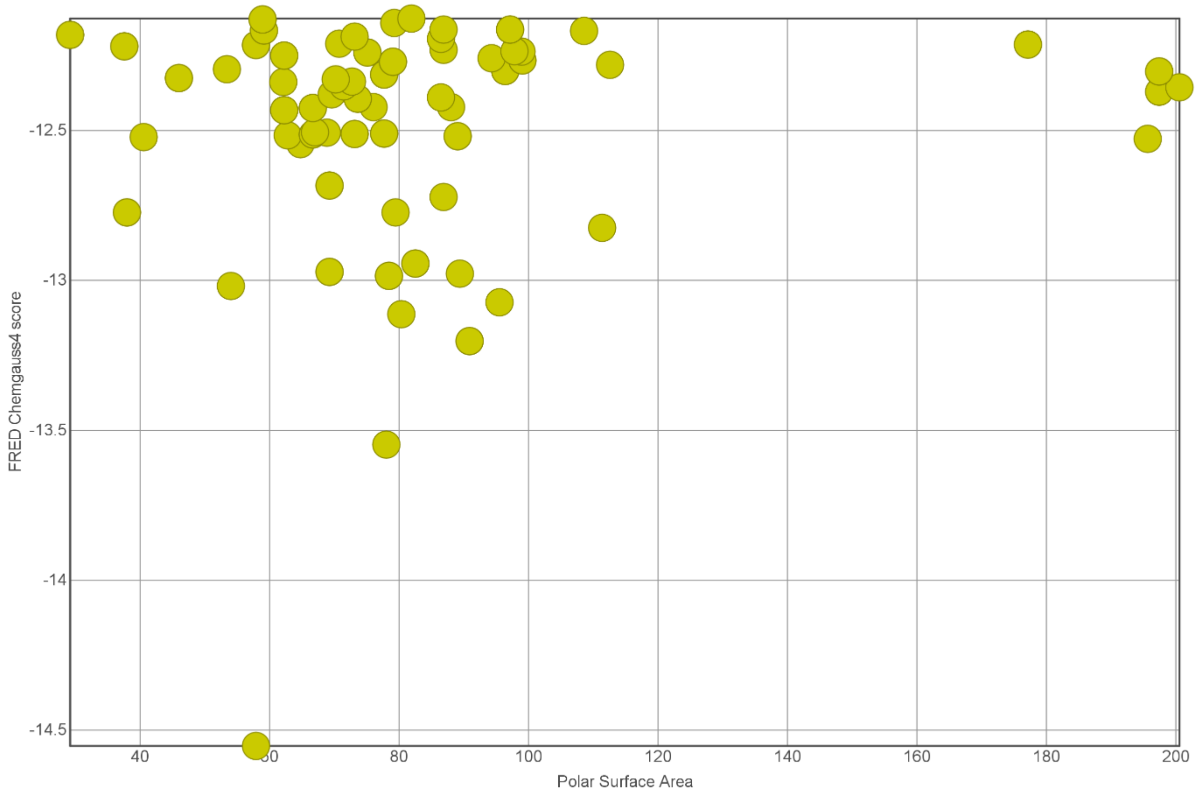

Figure S21: Compound PSA vs FRED Chemgauss4 score.

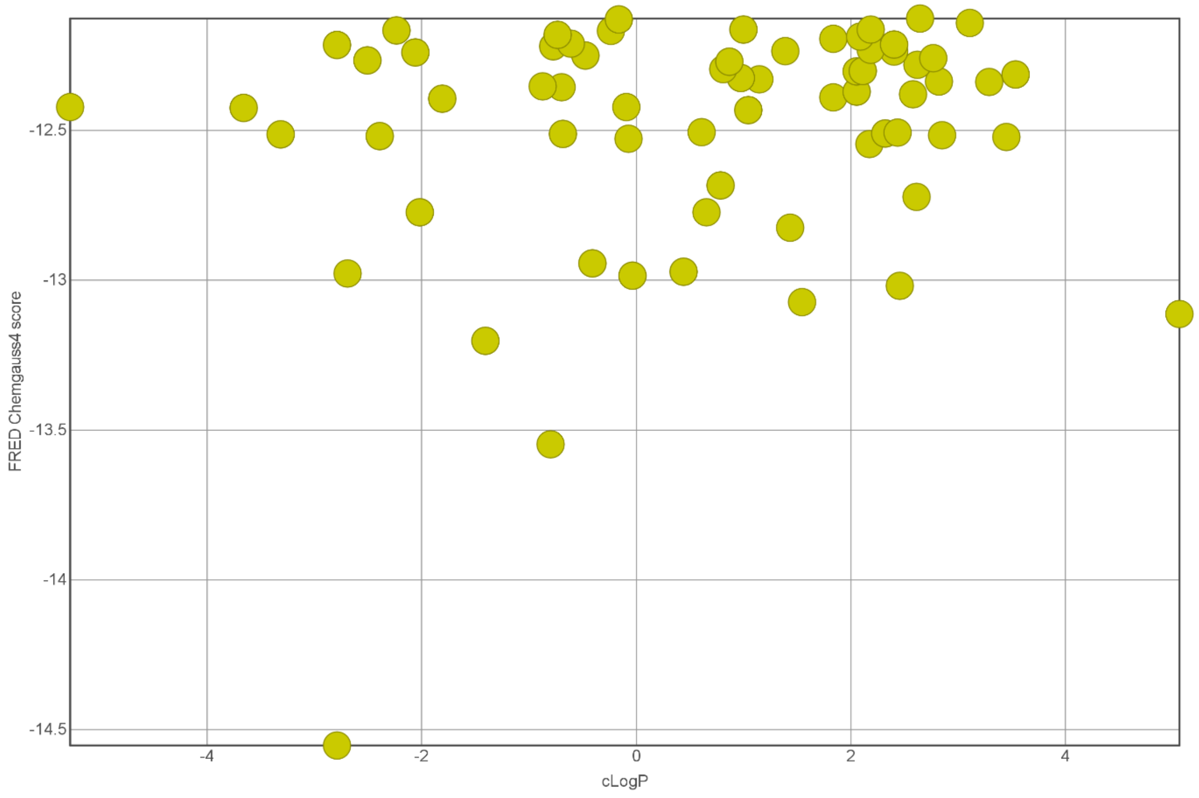

Figure S22: Compound cLogP vs FRED Chemgauss4 score.

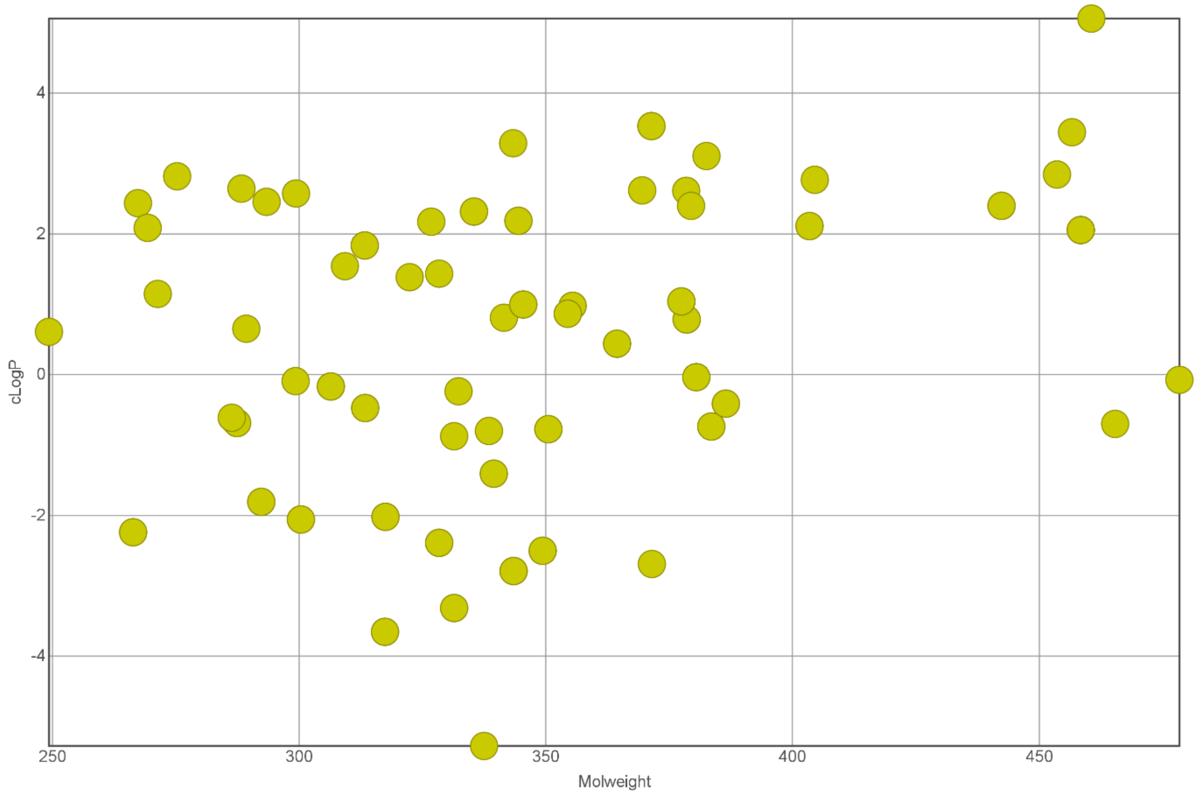

Figure S23: Compound MW vs compound cLogP.

9. Sequence alignment SARS-Cov and SARS-CoV-2 (Figure S24)

Query: 6LU7_1|Chain A|main protease|Severe acute respiratory syndrome coronavirus 2 (2697049)

Subject: 2QIQ_1|Chain A|Replicase polyprotein 1ab|SARS coronavirus (694009)

Score	Expect	Method	Identities	Positives	Gaps
612 bits(1578)	0.0	Compositional matrix adjust.	288/301(96%)	297/301(98%)	0/301(0%)

Query 1 SGFRKMAFPSGKVEGCMVQVTCGTTTLNGLWLDDVVYCPRHVICTSEDMLNPNYEDLLIR 60

+GFRKMAFPSGKVEGCMVQVTCGTTTLNGLWLDD VYCPRHVICT+EDMLNPNYEDLLIR

Sbjct 1 AGFRKMAFPSGKVEGCMVQVTCGTTTLNGLWLDDTVYCPRHVICTAEDMLNPNYEDLLIR 60

Query 61 KSNHNFLVQAGNVQLRVIGHSMQNCVLKLKVDTANPKTPKYKFVRIQPGQTFSVLACYNG 120

KSNH+FLVQAGNVQLRVIGHSMQNC+L+LKVDT+NPKTPKYKFVRIQPGQTFSVLACYNG

Sbjct 61 KSNHSFLVQAGNVQLRVIGHSMQNCLLRLKVDTSNPKTPKYKFVRIQPGQTFSVLACYNG 120

Query 121 SPSGVYQCAMRPNFTIKGSFLNGSCGSVGFNIDYDCVSFCYMHHMELPTGVHAGTDLEGN 180

SPSGVYQCAMRPN TIKGSFLNGSCGSVGFNIDYDCVSFCYMHHMELPTGVHAGTDLEG

Sbjct 121 SPSGVYQCAMRPNHTIKGSFLNGSCGSVGFNIDYDCVSFCYMHHMELPTGVHAGTDLEGK 180

Query 181 FYGPFVDRQTAQAAGTDTTITVNVLAWLYAAVINGDRWFLNRFTTTLNDFNLVAMKYNYE 240

FYGPFVDRQTAQAAGTDTTIT+NVLAWLYAAVINGDRWFLNRFTTTLNDFNLVAMKYNYE

Sbjct 181 FYGPFVDRQTAQAAGTDTTITLNVLAWLYAAVINGDRWFLNRFTTTLNDFNLVAMKYNYE 240

Query 241 PLTQDHVDILGPLSAQTGIAVLDMCASLKELLQNGMNGRTILGSALLEDEFTPFDVVRQC 300

PLTQDHVDILGPLSAQTGIAVLDMCA+LKELLQNGMNGRTILGS +LEDEFTPFDVVRQC

Sbjct 241 PLTQDHVDILGPLSAQTGIAVLDMCAALKELLQNGMNGRTILGSTILEDEFTPFDVVRQC 300

Query 301 S 301

S

Sbjct 301 S 301

2QIQ_1|Chain A|Replicase polyprotein 1ab|SARS coronavirus (694009)

Sequence ID: **Query_35561** Length: **301** Number of Matches: **1**

Range 1: 1 to 301 Graphics ▼ Next Match ▲ Previous Match

Score	Expect	Method	Identities	Positives	Gaps
612 bits(1578)	0.0	Compositional matrix adjust.	288/301(96%)	297/301(98%)	0/301(0%)

```
Query  1    SGFRKMAFPSGKVEGCMVQVTCGTTTLNGLWLDDVVYCPRHVICTSEDMLNPNYEDLLIR  60
            +GFRKMAFPSGKVEGCMVQVTCGTTTLNGLWLDD VYCPRHVICT+EDMLNPNYEDLLIR
Sbjct  1    AGFRKMAFPSGKVEGCMVQVTCGTTTLNGLWLDDTVYCPRHVICTAEDMLNPNYEDLLIR  60

Query  61   KSNHNFLVQAGNVQLRVIGHSMQNCVLKLKVDTANPKTPKYKFVRIQPGQTFSVLACYNG  120
            KSNH+FLVQAGNVQLRVIGHSMQNC+L+LKVDT+NPKTPKYKFVRIQPGQTFSVLACYNG
Sbjct  61   KSNHSFLVQAGNVQLRVIGHSMQNCLLRLKVDTSNPKTPKYKFVRIQPGQTFSVLACYNG  120

Query  121  SPSGVYQCAMRPNFTIKGSFLNGSCGSVGFNIDYDCVSFCYMHHMELPTGVHAGTDLEGN  180
            SPSGVYQCAMRPN TIKGSFLNGSCGSVGFNIDYDCVSFCYMHHMELPTGVHAGTDLEG
Sbjct  121  SPSGVYQCAMRPNHTIKGSFLNGSCGSVGFNIDYDCVSFCYMHHMELPTGVHAGTDLEGK  180

Query  181  FYGPFVDRQTAQAAGTDTTITVNVLAWLYAAVINGDRWFLNRFTTTLNDFNLVAMKYNYE  240
            FYGPFVDRQTAQAAGTDTTIT+NVLAWLYAAVINGDRWFLNRFTTTLNDFNLVAMKYNYE
Sbjct  181  FYGPFVDRQTAQAAGTDTTITLNVLAWLYAAVINGDRWFLNRFTTTLNDFNLVAMKYNYE  240

Query  241  PLTQDHVDILGPLSAQTGIAVLDMCASLKELLQNGMNGRTILGSALLEDEFTPFDVVRQC  300
            PLTQDHVDILGPLSAQTGIAVLDMCA+LKELLQNGMNGRTILGS +LEDEFTPFDVVRQC
Sbjct  241  PLTQDHVDILGPLSAQTGIAVLDMCAALKELLQNGMNGRTILGSTILEDEFTPFDVVRQC  300

Query  301  S  301
            S
Sbjct  301  S  301
```

Figure S24: Alignment of SARS-Cov and SARS-CoV-2 sequence.

SUPPORTING INFORMATION REFERENCES

[1] Wang, R.; Ying, F.; Lai, L. A new atom-additive method for calculating partition coefficients, *J. Chem. Inf. Comput. Sci.*, **1997**, 37, 615-621.

[2] McGovern S.L.; Helfand, B.T.; Feng, B.; Shoichet, B.K. A Specific Mechanism of Nonspecific Inhibition, *J. Med. Chem.*, **2003**, 46, 4265-4272.

[3] Seidler J.; McGovern, S.L.; Doman, T.N.; Shoichet, B.K. Identification and Prediction of Promiscuous Aggregating Inhibitors Among Known Drugs, *J. Med. Chem.*, **2003**, 46, 4477-4486.

[4] Konc, J.; Miller, B. T.; Štular, T.; Lešnik, S.; Woodcock, H. L.; Brooks, B. R., Janežič, D. ProBiS-CHARMMing: web interface for prediction and optimization of ligands in protein binding sites. *J. Chem. Info. Model.* **2015**, *55*, 2308−2314.

[5] Konc, J.; Česnik, T.; Konc, J. T.; Penca, M.; Janežič, D. ProBiS-database: precalculated binding site similarities and local pairwise alignments of PDB structures. *J. Chem. Info. Model*. **2012**, *52(2)*, 604-612.

[6] Konc, J.; Janežič, D. ProBiS tools (algorithm, database, and web servers) for predicting and modeling of biologically interesting proteins. *Prog. Biophys. Mol. Biol*. **2017**, *128*, 24-32.

[7] McGann M. FRED and HYBRID docking performance on standardized datasets. *J. Comp.-Aid. Mol. Design.* **2012**, *26(8)*, 897–906.

[8] McGann M. FRED Pose Prediction and Virtual Screening Accuracy. *J. Chem. Inf. Model.* **2011**, 51(3), 578–596.

[9] McGann M., Almond H.R., Nicholls A., Grant J.A., Brown F.K. Gaussian docking functions. *Biopolymers* **2003**, *68(1)*, 76.

[10] Jamroz, M., Kolinski, A. ClusCo: clustering and comparison of protein models. *Bmc Bioinformatics* **2013**, *14(1)*, 1-6.

[11] Theobald, D. L., Wuttke, D. S. THESEUS: maximum likelihood superpositioning and analysis of macromolecular structures. *Bioinformatics* **2006**, *22(17)*, 2171-2172.
